# Activatable NIR-II organic fluorescent probes for bioimaging

**DOI:** 10.7150/thno.71359

**Published:** 2022-04-11

**Authors:** Xiaoning Zhang, Shasha Li, Huizhen Ma, Hao Wang, Ruiping Zhang, Xiao-Dong Zhang

**Affiliations:** 1 Tianjin Key Laboratory of Brain Science and Neural Engineering, Academy of Medical Engineering and Translational Medicine, Tianjin University, Tianjin 300072, China.; 2 Department of Physics and Tianjin Key Laboratory of Low Dimensional Materials Physics and Preparing Technology, School of Sciences, Tianjin University, Tianjin 300350, China.; 3 The Third Hospital of Shanxi Medical University, Shanxi Bethune Hospital, Taiyuan, 030000, China.

**Keywords:** NIR-II fluorescence, organic fluorescent probes, responsive probes, pathological changes, bioimaging

## Abstract

NIR-II imaging is developed rapidly for noninvasive deep tissue inspection with high spatio-temporal resolution, taking advantage of diminished autofluorescence and light attenuation. Activatable NIR‐II fluorescence probes are widely developed to report pathological changes with accurate targeting, among which organic fluorescent probes achieve significant progress. Furthermore, the activatable NIR‐II fluorescent probes exhibited appealing characteristics like tunable physicochemical and optical properties, easy processability, and excellent biocompatibility. In the present review, we highlight the advances of activatable NIR-II fluorescence probes in design, synthesis and applications for imaging pathological changes like reactive oxygen species (ROS), reactive nitrogen species (RNS), reactive sulfur species (RSS), pH, hypoxia, viscosity as well as abnormally expressed enzymes. This non-invasive optical imaging modality shows a promising prospect in targeting the pathological site and is envisioned for potential clinical translation.

## Introduction

Fluorescence imaging in the second near-infrared window (NIR-II, 1000-1700 nm) is developed rapidly for noninvasive deep tissue investigation with high spatio-temporal resolution due to diminished autofluorescence light attenuation 1-8. For optical *in vivo* bioimaging, the penetration depth of photons is mainly influenced by the absorption and scattering of tissue components. Meanwhile, the autofluorescence from the tissue will produce background noise. Compared with the traditional near-infrared window (NIR-I,750-900 nm), the NIR-II fluorescence has reduced photon scattering and diminished autofluorescence taking advantage of its long wavelength, which allows the photon to penetrate deeper biological tissue and improves the spatial resolution. Therefore, a variety of NIR-II fluorescence probes are exploited for biosensing and bioimaging 9-21. Thus far, a wide variety of materials especially inorganic nanostructures have been applied in fluorescence imaging 22-24, such as carbon nanotubes 24-26, quantum dots (QDs) 27-29, and lanthanide-doped nanoparticles (LnNPs) 30, 31, which may arise safety concern about cumulative toxicity. Organic fluorescent probes offer a better alternative due to their tunable physical and optical properties controlled by structural engineering, easy processability, and excellent biocompatibility 32-39.

NIR-II fluorescence probes show potential in cancer imaging and diagnosis 40, medical detection 8, and vascular bioimaging 41. It is worth noting that, repurposing NIR-I probes with emission in the NIR-II window, especially the ones approved clinically, provides an important development strategy for NIR-II fluorescence proves to accelerate the clinical translation 42. Organic small molecules and polymers are the main forms of organic fluorescent probes. Organic small molecules are mostly designed based on the D-A-D architectures, cyanine, and boron dipyrromethene (BODIPY) scaffold, and derived multiple imaging agents and functional fluorescent probes with high biocompatibility, fast excretion, and superior optical properties. However, low quantum yield (QY), short emission wavelength, and time-consuming synthesis and purification process, still limit their further bioimaging application. Polymers are mostly designed based on a-electron delocalized backbones, and attract attentions to develop fluorescent probes with superior optical properties and adjustable structure. However, improving the biocompatibility and accelerating the excretion are still challenges. To date, researchers have made great progress on the development of organic fluorescent probes, and organic fluorescence probes showed promising prospects on bioimaging.

Most of the fluorescence probes are “always-on” probes, which improve the target-to-background ratio (TBR) by accumulation and retention in the target tissues. However, it may produce more false-positive signals with no specificity and reduce the imaging effect. While activatable probes produce strong signals only under the specific biological alterations compared with the “always-on” probes. These alterations are usually associated with pathological changes in the target site such as abnormal redox, pH, enzyme expression, etc., which makes the activatable probes more accurate for specific disease diagnoses 43-46.

Multiple “turn-on” fluorescence probes including nanostructures in NIR-I and NIR-II windows were reported and reviewed 47, 48, and in the present review, we highlight the up-to-date advances of activatable organic NIR-II fluorescence probes in design, synthesis and application for the detection of ROS, RNS, RSS, pH, viscosity and enzyme. This state-of-the-art, non-invasive and highly specific optical imaging modality shows promising prospects in understanding the fundamental pathophysiology of diseases and is envisioned for potential clinical translation.

## Redox species-activatable probes

Redox homeostasis plays a critical role in maintaining cellular function related to necessary physiological activities, and redox imbalance is related to the occurrence and development of multiple diseases such as cancer 49, 50. Important redox species in the living organism include ROS, RNS, and RSS. ROS and RNS can be generated through exogenous and endogenous sources. Exogenous sources may include UV radiations, γ-radiations, drugs, food, toxic chemicals, etc. The endogenous sources may include direct producing enzymes, indirect producing enzymes, neutrophils, cytokines and other components of white blood cells, mitochondrial, metals and side effects of various diseases. These molecules interact with each other to engage in important physiological activities, and multiple clinical disorders are associated with the early initiation of these important signaling molecules. RSS plays the function of regulating protein catalytic activity through polysulfidation, the formation of polysulfides [-S-(S)n-H] on their reactive cysteine residues. Both ROS and RNS can lead to cellular dysfunction via lipid peroxidation, protein and DNA damage when present in high concentrations. Therefore, monitoring the ROS, RNS, and RSS provides a powerful tool for related disease diagnoses such as Parkinson's disease and Alzheimer's disease 51-54.

**Figure 1:**
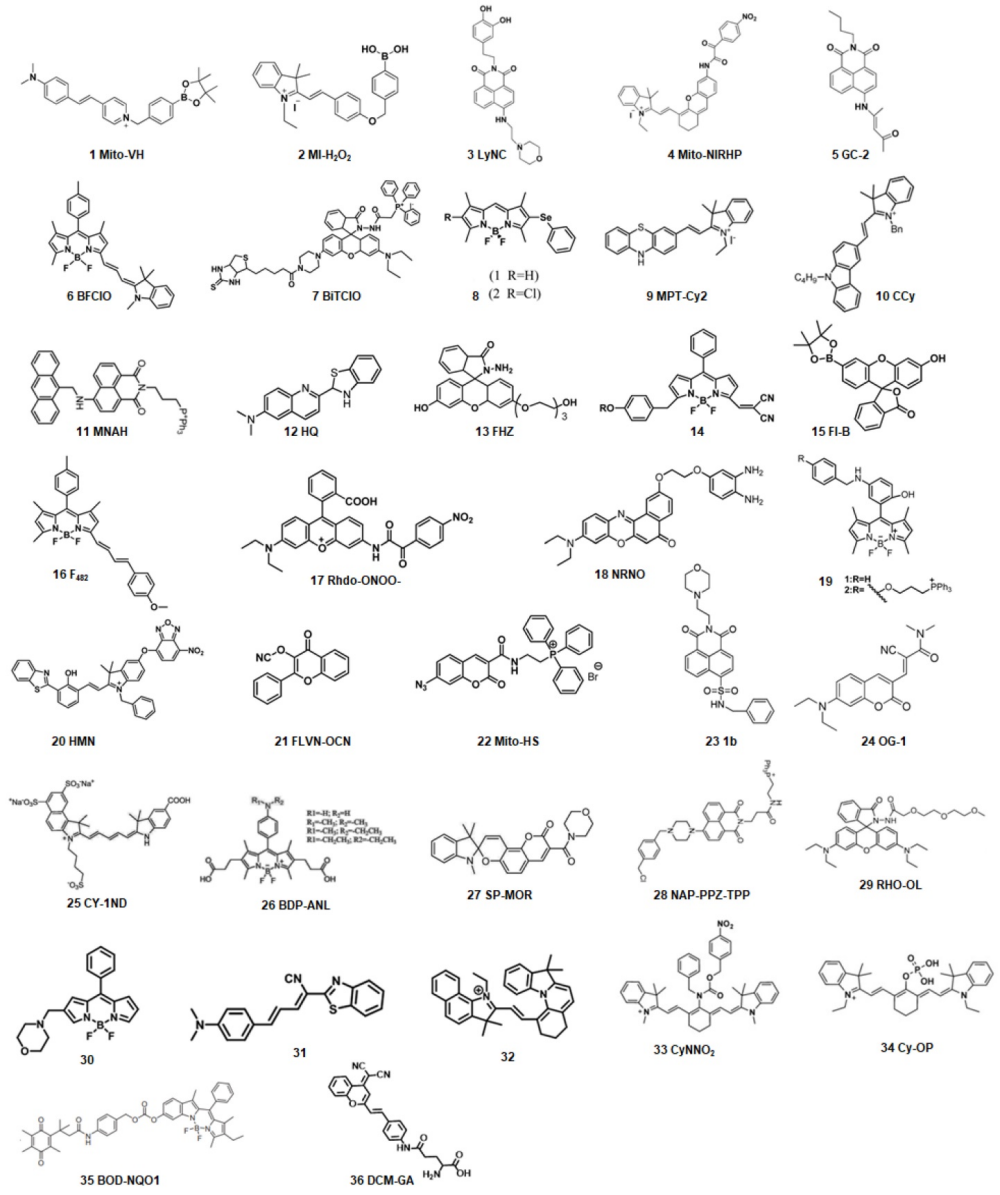
The representative activatable NIR-II organic fluorescent probes. Detecting ROS (probes 1-13), RNS (probes 14-19), RSS (probes 20-24), pH (probes 25-29), viscosity (probes 30-32), enzymes (probes 33-36).

**Figure 2:**
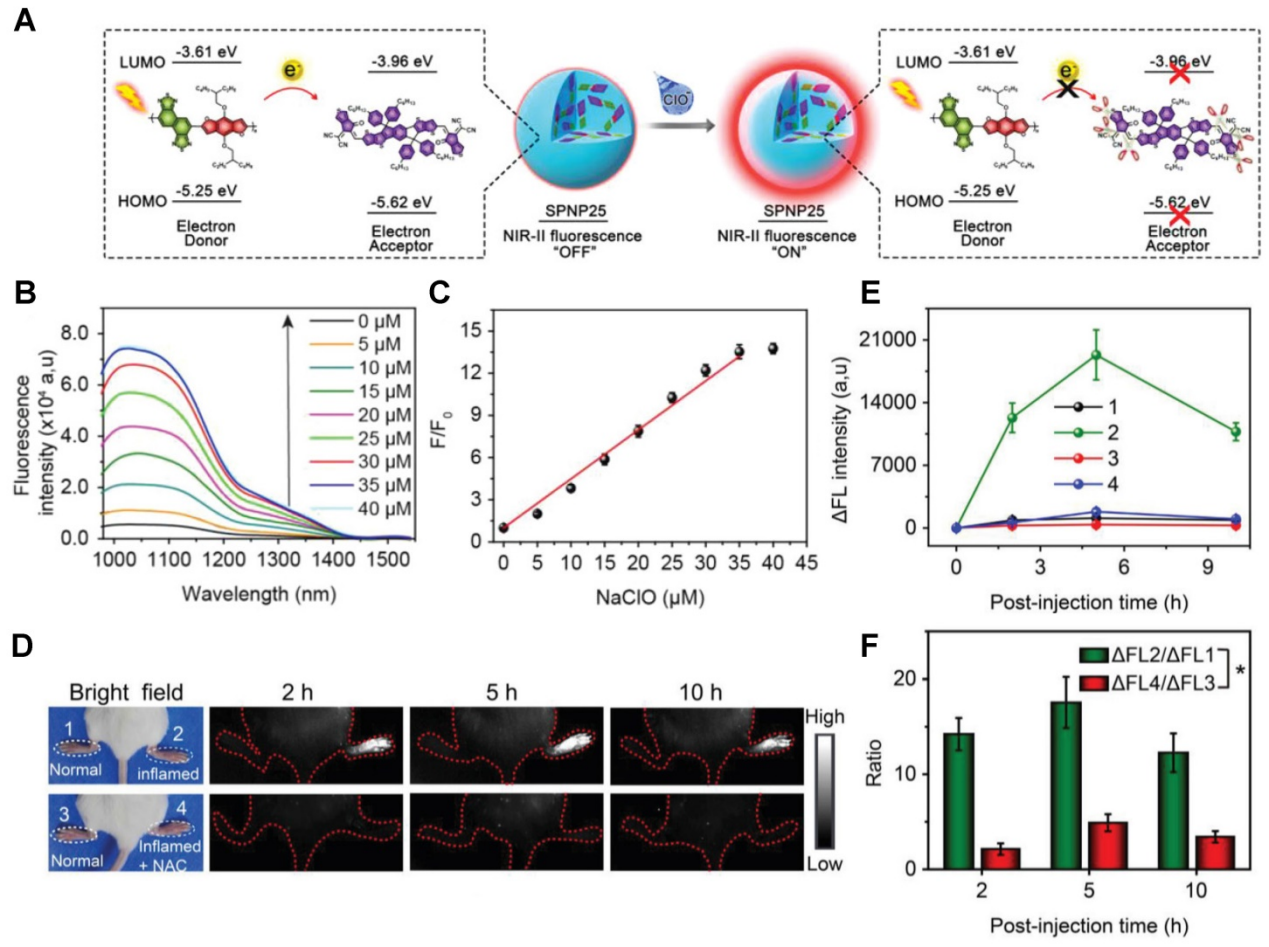
** Activatable NIR-II probes for ClO^-^. (A)** The structure and detection mechanism of SPNP25 for ClO^-^ by blending the hydrophobic donor PDF and acceptor ITTC. **(B)** Fluorescence spectra of the nanoprobe SPNP25 treated with ClO^-^ (0-40 µM). **(C)** Plots of the linear relationship between fluorescence intensity and concentrations of ClO^-^. **(D)** Real-time NIR-II fluorescence images of SPNP25 in LPS-pretreated and LPS/NAC-treated mice (Excitation: 808 nm laser, 40 mW cm^-2^). **(E)** Corresponding fluorescence intensity of the region of interest after administration of SPNP25.** (F)** The NIR-II fluorescence intensity enhancement of the ratio in different regions of interest. Figures adapted with permission from 72. Copyright © 2018, John Wiley and Sons.

**Figure 3:**
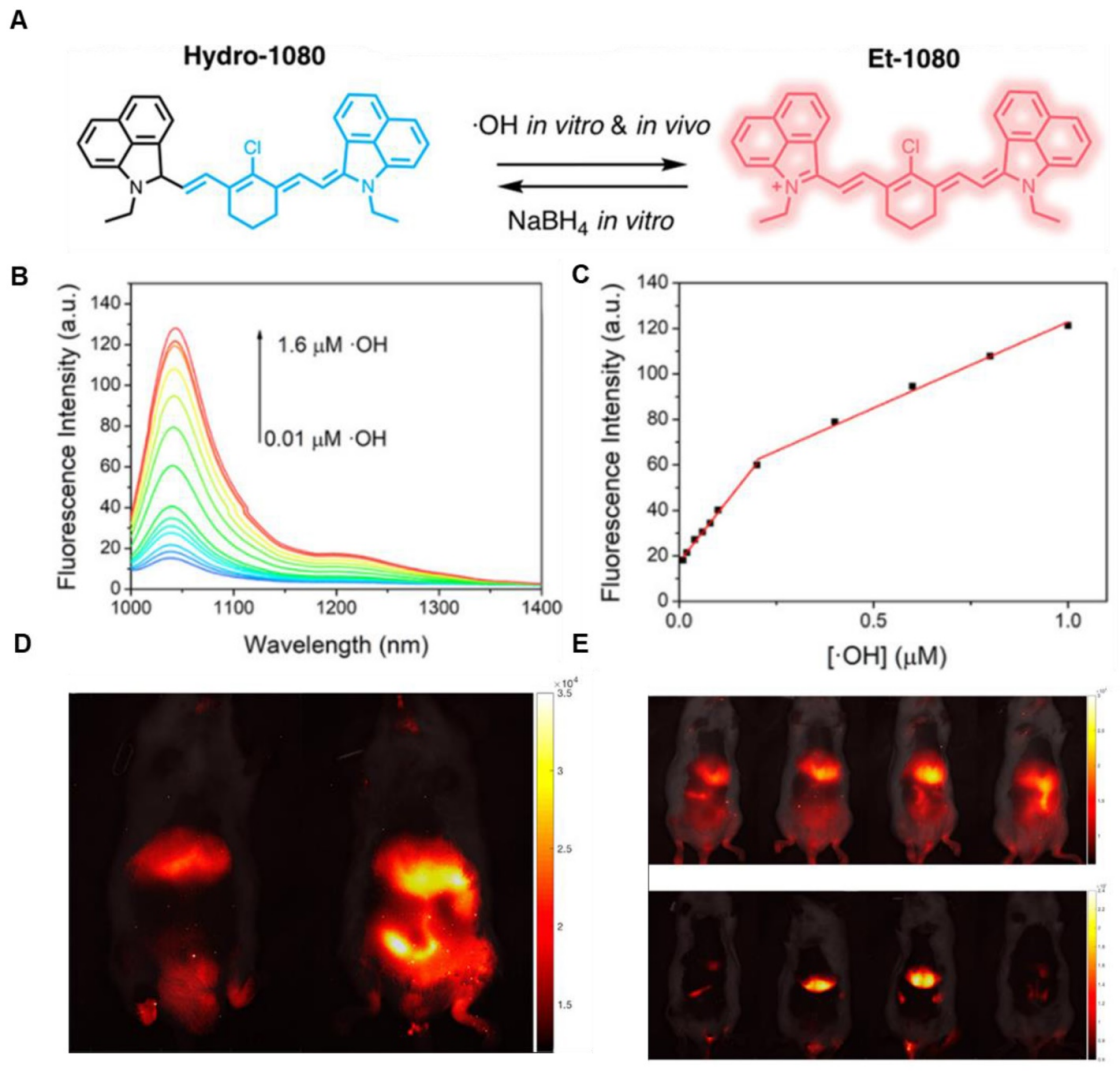
** Activatable NIR-II probes for •OH. (A)** The structure and detection mechanism of Hydro-1080 for •OH. **(B)** NIR-II fluorescence spectra of Hydro-1080 treated with •OH (0.01-1.6 µM) under 980 nm excitation. **(C)** Plots of the linear relationship between fluorescence intensity at 1044 nm and concentrations of •OH in the range of 10-200 and 200-1000 nM. **(D)** NIR-II fluorescence images of mice injected with saline and LPS and intravenously injected with Hydro-1080 after 24 h. **(E)** NIR-II and NIR-IIa fluorescence images of mice injected with 0, 300, 500, 500 mg/kg APAP, respectively, and then intravenously injected with Hydro-1080. The last group is pretreated with inhibitor ABT. Figures adapted with permission from 73. Copyright © 2019 American Chemical Society.

**Figure 4:**
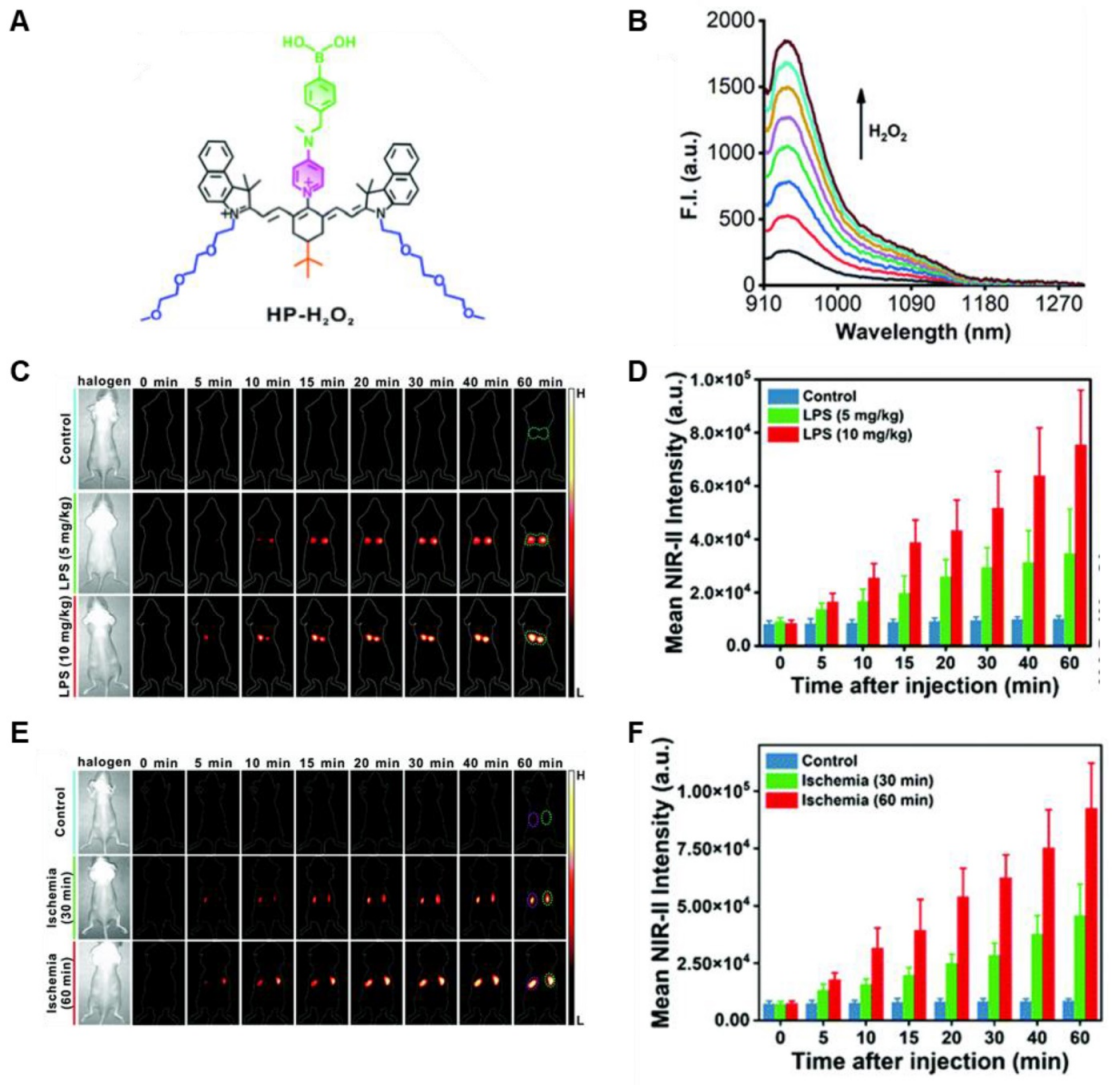
** Activatable NIR-II probes for H_2_O_2_. (A)** The structure of HP-H_2_O_2_ for detecting H_2_O_2_. **(B)** Fluorescence spectra of HP-H_2_O_2_ (10 µM) upon addition of ONOO^-^ (0-100 µM). **(C)** Real-time NIR-II fluorescence imaging of healthy mice and ALI mice after intratracheal instillation of HP-H_2_O_2_ (Excitation: 808 nm laser, 40 mW cm^-2^). **(D)** Mean NIR-II fluorescence intensities of regions of interest in **(C)**. **(E)** Real-time NIR-II fluorescence imaging of healthy mice and AKI mice after intravenous administration of HP-H_2_O_2_ (Excitation: 808 nm laser, 40 mW cm^-2^). **(F)** Mean NIR-II fluorescence intensities of regions of interest in **(E)**. Figures adapted with permission from 74. Copyright © 2022, Royal Society of Chemistry.

**Figure 5:**
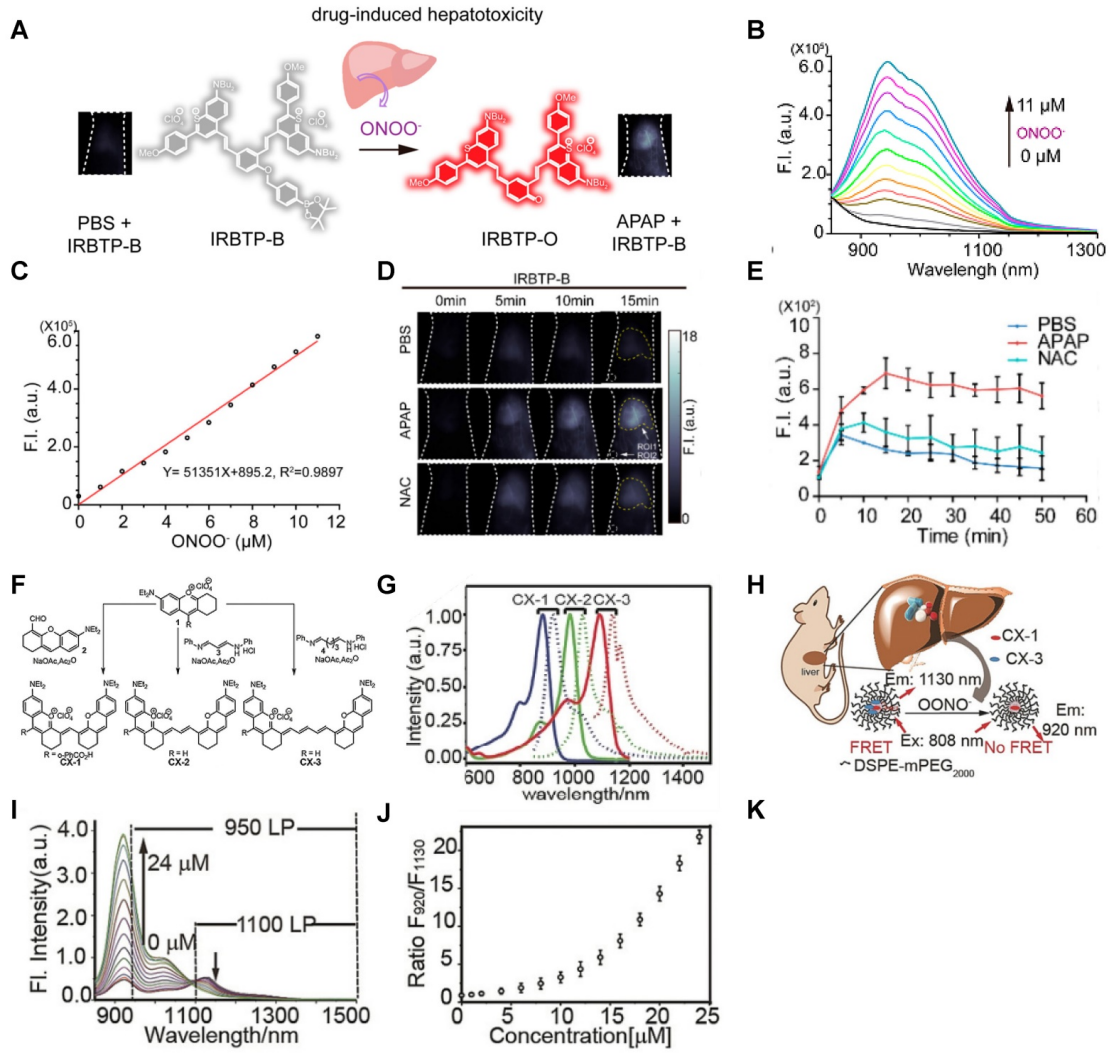
** Activatable NIR-II probes for ONOO^-^. (A)** The structure and mechanism of IRBTP-B. **(B)** Fluorescence spectra of IRBTP-B (10 µM) treated with ONOO^-^ (0-11 µM). **(C)** Plots of the linear relationship between fluorescence intensity at 950 nm and concentrations of ONOO^-^. **(D)** Real-time NIR-II fluorescence imaging of livers of mice from IRBTP-B treated with PBS, APAP, and NAC + APAP respectively. **(E)** Relative fluorescence intensity of livers of mice treated with various substances followed by IRBTP-B over time. **(F)** The synthesis of CX dyes. **(G)** Corresponding absorption (solid) and emission (dot) spectra of CX dyes. **(H)** The structure and mechanism of IRBTP-O, which is constructed by loading CX-1 and CX-3 into a micelle. **(I)** Fluorescence spectra of PN1100 in the presence of ONOO^-^. **(J)** The ratio of F_920_/F_1130_ (fluorescence intensity at 920 nm and 1130 nm, respectively) of PN1100 upon addition with OONO^-^ (0-24 µm). **(K)** The ratio of F_950_/F_1100_ of the livers of mice treated with PBS, APAP, and NAC + APAP respectively, followed by PN1100 over time. Figures adapted with permission from 83, 84. Copyright © 2019, American Chemical Society, and Copyright © 2019, John Wiley and Sons.

**Figure 6:**
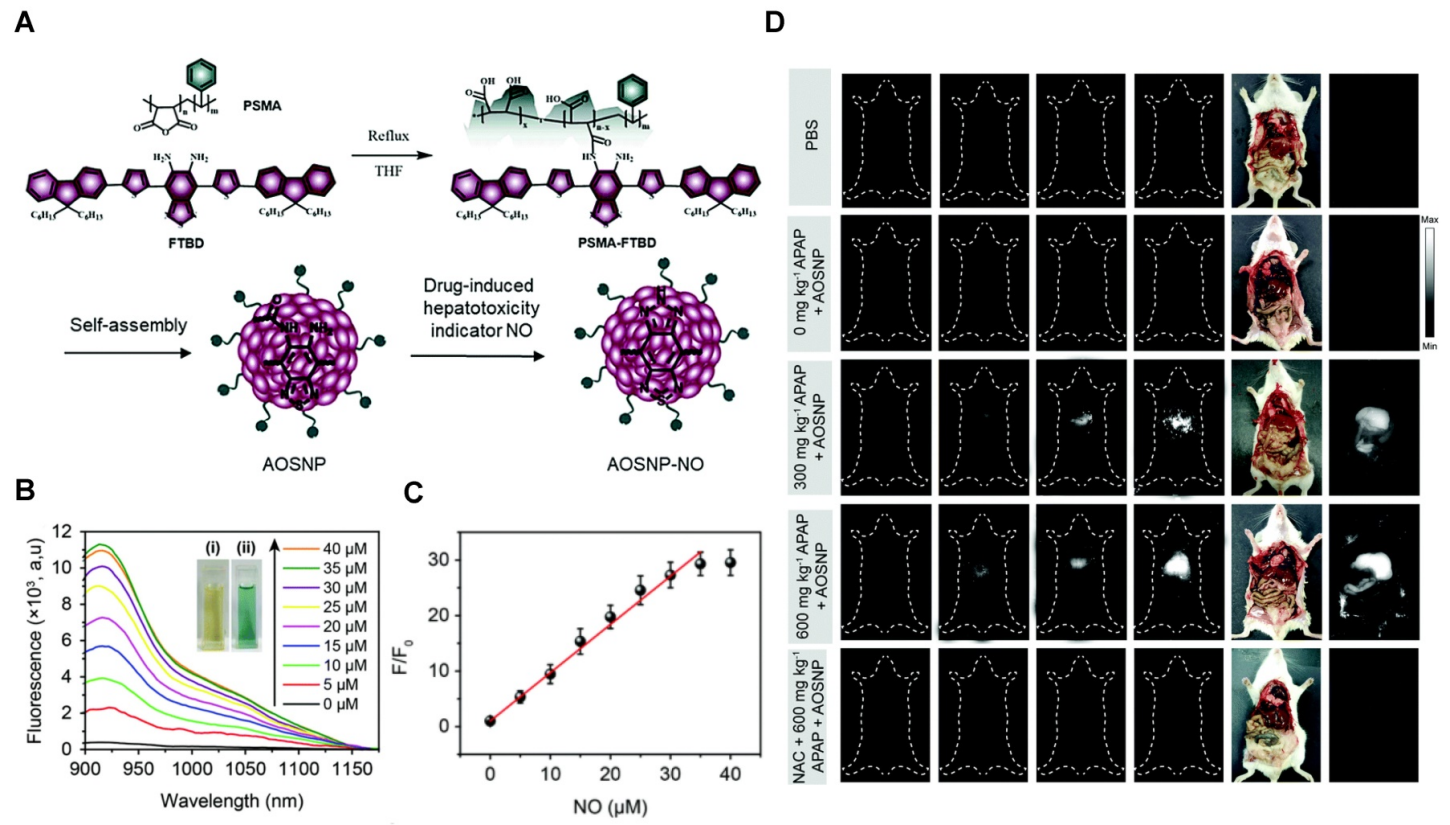
** Activatable NIR-II probes for NO. (A)** The structure and mechanism of AOSNP. **(B)** Fluorescence spectra of the AOSNP (3 µg mL^-1^) treated with various concentrations of NO. Insets show photographs of the AOSNP i before and ii after the addition of NO.** (C)** Plots of the linear relationship between fluorescence intensity (F/F_0_, 1000-1700 nm) and intensity and concentrations of NO (0-35 µM).** (D)** *In vivo* images of dose-dependent hepatotoxicity in mice given APAP. Representative images of the control mice treated with PBS or APAP (0 mg kg^-1^), representative images of mice at various time points after receiving AOSNP (100 µL, 10 mg mL^-1^) pre-treated with 300 mg kg^-1^ APAP, 600 mg kg^-1^ APAP, 300 mg kg^-1^ NAC followed by 600 mg kg^-1^ APAP. Figures adapted with permission from 85. Copyright © 1996, Royal Society of Chemistry.

**Figure 7:**
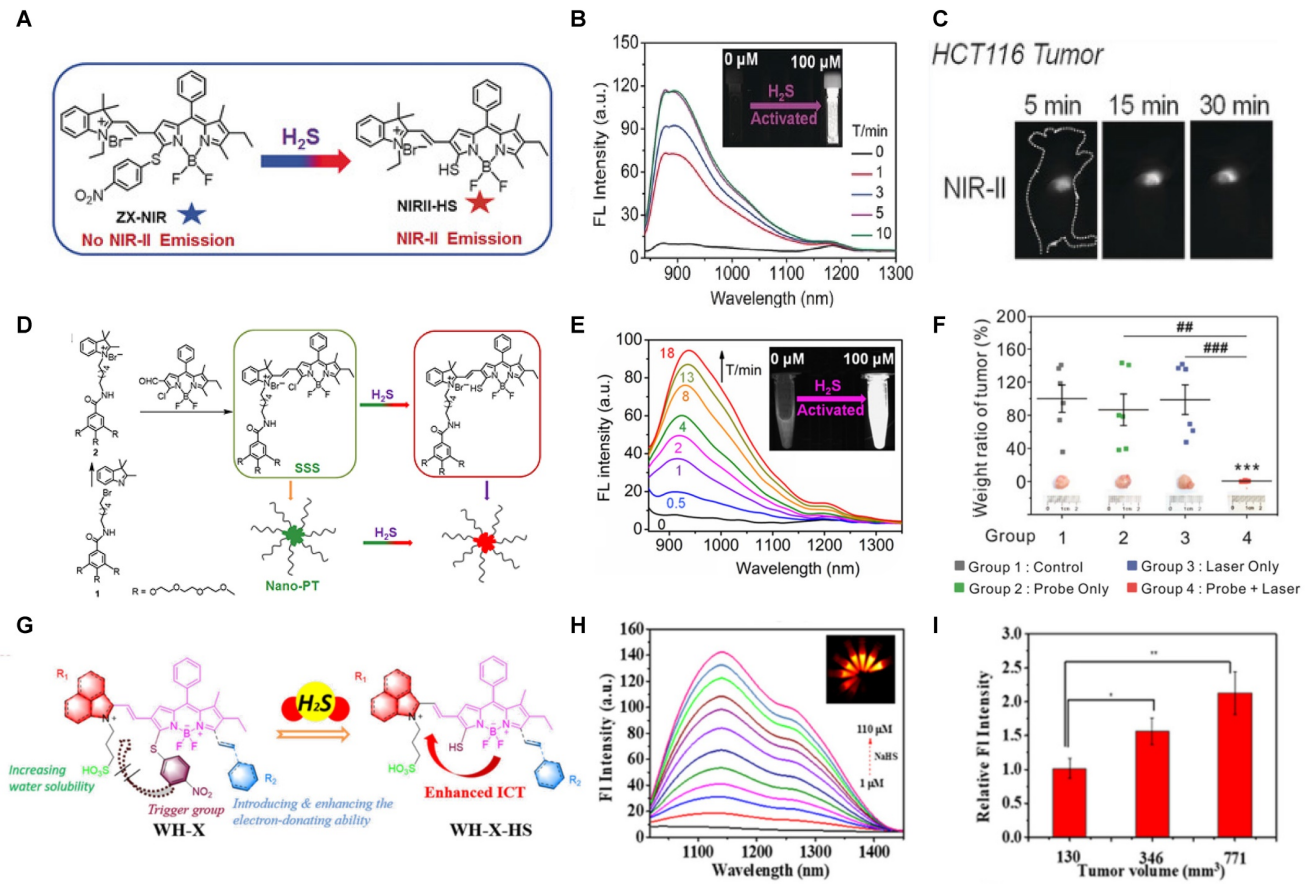
** Activatable NIR-II probes for H_2_S. (A)** The fabrication and activation process of probe NIR-II@Si for H_2_S, based on trapping ZX-NIR and aza-BOD into the hydrophobic interior of self-assembled micellar aggregates. **(B)** NIR-II emission spectra of NIR-II@Si (10 µm ZX‐NIR) in the presence of 100 µm NaHS over time, λex = 780 nm. Insets show photographs of the NIR-II@Si before and after the addition of NaHS. **(C)** Real-time images of cancers after subcutaneously injecting the NIR-II@Si (20 nmol ZX-NIR) into the tumor regions of HCT116 tumor-bearing mice. **(D)** The fabrication and activation process of Nano-PT, which is based on the self-assembly of an H_2_S-responsive small molecule capable of excellent photothermal conversion efficiency. **(E)** Time-dependent NIR-II emission spectra of Nano-PT (20 µM SSS) treated with 100 µM NaHS, λex = 790 nm. Inset shows photographs of the H_2_S-activated NIR-II emission. **(F)** Ratio of tumor weight of HCT116 tumor-bearing mice in different groups relative to that in untreated mice and photographs of tumor tissues. **(G)** The structure of WH, based on BODIPY skeleton and 4-Nitrothiophenol for specific recognition site.** (H)** NIR-II fluorescence spectra of WH-3 in the presence of NaHS (1-110 µM). Inset shows photographs of the NIR-II emission activated by varying NaHS. **(I)** Relative fluorescence intensity of different-sized tumor mice treated with the WH-3. Figures adapted with permission from 86, 93, 94. Copyright © 2018, John Wiley and Sons, Copyright © 2018, and Copyright © 2021, American Chemical Society.

**Figure 8:**
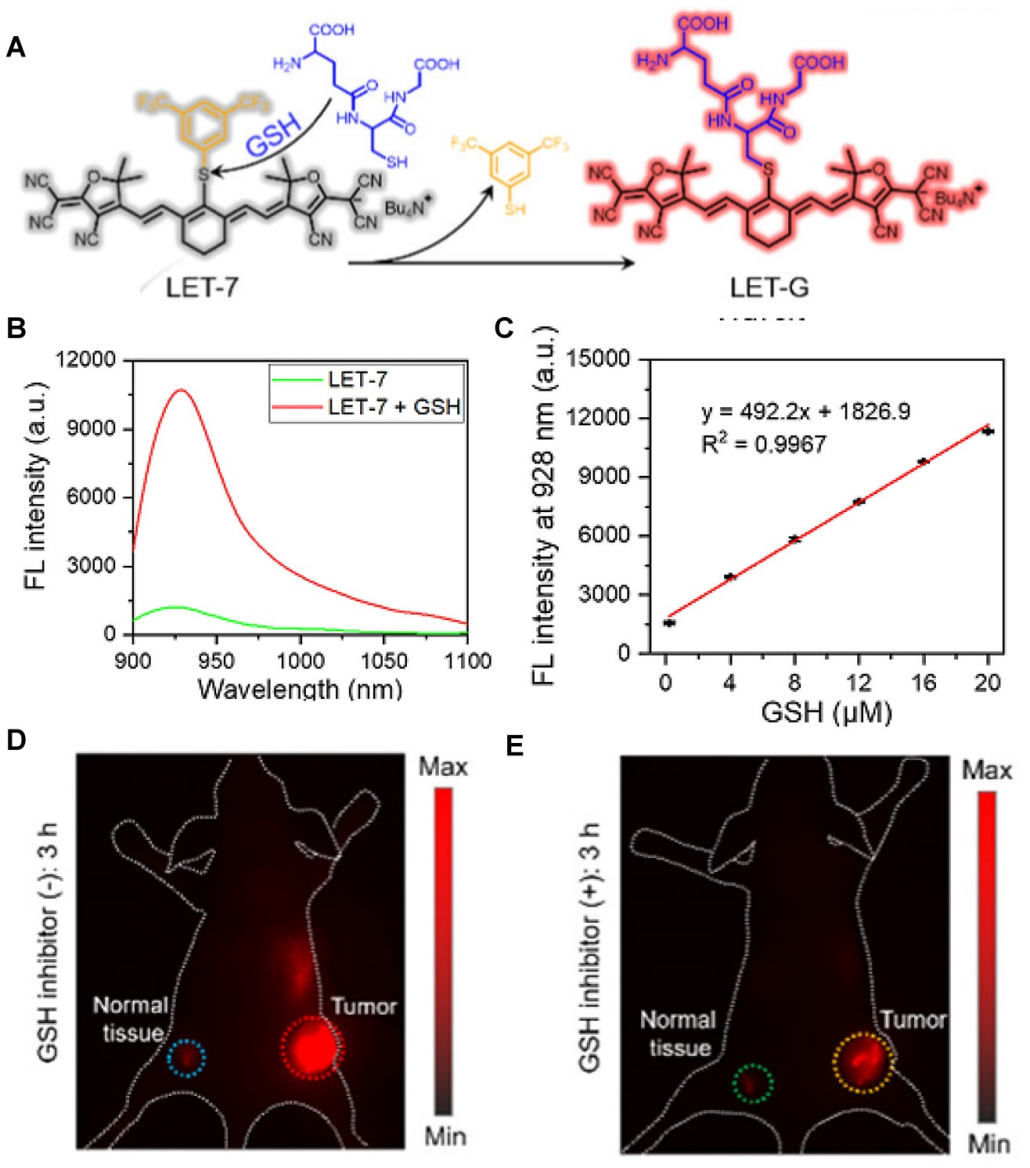
** Activatable NIR-II probes for GSH. (A)** The structure and mechanism of LET-7. **(B)** The fluorescence spectra of the LET-7 with or without GSH. **(C)** The plot of the linear relationship between fluorescence intensity at 928 nm and concentrations of GSH. NIR-II images of tumor-bearing mice, 3 h after administration of LET-7 without **(D)** and with **(E)** GSH inhibitor treatment. Figures adapted with permission from 95. Copyright © 2021 American Chemical Society.

In the past decades, many redox-responsive fluorescent probes have been developed 55-57, among which organic fluorescent probes have been widely investigated owing to good biocompatibility and easily tunable structure and optical properties (**Figure 1, Table 1 and Figure 2-8**). Probes are designed based on the reactivities of the redox species and fluorescence mechanisms such as photon-induced electron transfer (PET), intramolecular charge transfer (ICT), excited-state intra molecular proton transfer (ESIPT), and fluorescence resonance energy transfer (FRET). For instance, to detect ROS, reductive groups like lactam and double bond were adopted to react with the analyte and make the probes emit fluorescence. o-Phenylenediamine is commonly used to form triazole with nitric oxide for inhibiting PET of the probe and making it fluorescent. The nucleophilicity of the RSS is utilized to design the activatable probes that can have addition and thiolytic reaction with RSS and become fluorescent.

### ROS-activatable probes

ROS are produced in necessary physiological processes. ROS with high reactivity consists of singlet oxygen (^1^O_2_), superoxide anion (O_2_^•-^), hydrogen peroxide (H_2_O_2_), hydroxyl radical (•OH), hypochlorous acid/hypochlorite (HOCl/^-^OCl), hypobromous acid/hypobromite (HOBr/^-^OBr) and peroxyl radical (ROO•) 52, 54, 58. While excess ROS can induce oxidative stress and damage tissues and cells, demonstrating a crucial pathogenic mechanism in conditions such as inflammatory diseases, cancers, and neurodegenerative disorders. Recently, a variety of probes have been developed for the detection of ROS, providing tools for early disease detection. Additionally, the structure-activity relationship between fluorescence probes structure and ROS have been revealed and can be used to guide the design of new probes.

As one of the most common ROS, H_2_O_2_ is involved in various inflammatory diseases and it can serve as an oxidative stress biomarker. The sensing strategies of H_2_O_2_-activatable probes include the reaction of aryl boronates or phenylboronic acid to phenols (probe 1, 2), oxidation of phenol to quinone (probe 3), the conversion of oxonium or diketone to acid (probe 4), and the cleavage of carbon-carbon double bond (probe 5) 59-63. In the presence of H_2_O_2_, the probes turn into corresponding reaction products, causing changes in fluorescence properties.

HOCl plays an essential role in the immune system *in vivo*. Endogenous HOCl is mainly generated from H_2_O_2_ and chloride ions (Cl^-^) catalyzed by the heme enzyme myeloperoxidase (MPO) in the neutrophils and exist as a mixture of the undissociated acid and the hypochlorite ion at physiologic pH levels. The over-production of HClO/ClO^-^ can be the biomarker of some ROS-related diseases. Based on the strong oxidation ability of HClO/ClO^-^, the sensing strategy of HClO/ClO^-^-activatable probes is to adopt oxidable building blocks like double bonds (probe 6), lactam or lactone (probe 7), thioether or selenide (probe 8) and so on 64-66.

•OH is widely associated with various pathological processes, and the detection of •OH *in situ* and *in vivo* is vital to understand these mechanisms. Based on the strong oxidation ability of •OH, the sensing strategies of •OH-activatable probes are designed on the oxidation of phenothiazines (probe 9) and the pyrrolidine (probe 10) 67, 68. Other ROS like ^1^O_2_, O_2_^•-^, HOBr/^-^OBr has similar sensing strategies based on their specific natures (probe 11, 12, 13) 69-71.

In 2018, a ClO^-^-activatable NIR-II fluorescence probe SPNP25 was developed by blending a ClO^-^-sensitive organic semiconducting non-fullerene acceptor (ITTC) with a ClO^-^-inert semiconducting polymer donor (PDF) in an amphiphilic-polymer-coated single nanoparticle (**Figure 2A-C**). This is based on a fairly generic bio-erasable intermolecular donor-acceptor interaction strategy. The specific response to ClO^-^ is achieved by degradation of ITTC in the presence of ClO^-^, thus erasing the intermolecular donor-acceptor interaction and uncaging NIR-II fluorescence of PDF. The *in vivo* NIR-II fluorescence imaging of ClO^-^ in an inflamed mouse model further confirms the specific response to ClO^-^ of SPNP25 (**Figure 2D-E**) 72. This smart design strategy changes the fluorescence intensity utilizing the oxidative cleavage reaction by ClO^-^. While the probe still needs to be modified like reducing the size of SPNP25 to improve biocompatibility and improving QY for further clinical translation.

In 2019, an •OH-activatable NIR-II fluorescence probe Hydro-1080 was designed based on direct breaking/recovering the conjugated system and rigid planar structure, which was successfully applied to imaging overproduced •OH of hepatotoxicity (**Figure 3A**). In the presence of •OH, Hydro-1080 turns on its NIR-II emission at 1044 nm due to the recovered conjugated system. Hydro-1080 shows a perfect linear relationship with the concentration of •OH, which provides a tool for related diseases imaging (**Figure 3B-C**). The results show the successful imaging of overproduced •OH on the liver injury model induced by lipopolysaccharide (LPS) and Acetaminophen (APAP) compared with the control group. Furthermore, Hydro-1080 holds the application potential in the NIR-IIa imaging, with high signal-to-background ratios up to 6.0 (**Figure 3D-E**) 73. This reduction synthesis route provides more bright ideas for the development of activatable NIR-II fluorescence probes. Hydrophobicity and QY of Hydro-1080 are the challenges of further application.

In 2021, an H_2_O_2_-activatable NIR-II fluorescence probe HP-H_2_O_2_ was designed based on the stable heptamethine-cyanine HP-N dyes, introducing phenylboronic acid as an H_2_O_2_-responsive element and fluorescence quencher (**Figure 4A**). HP-H_2_O_2_ shows increasing fluorescence intensity in the presence of H_2_O_2_ with increased concentration (**Figure 4B**). In the presence of H_2_O_2_, HP-H_2_O_2_ amplifies its NIR-II fluorescence intensity due to fast photoinduced electron transfer from cleavage of phenylboronic acid. Meanwhile, HP-H_2_O_2_ shows a perfect linear relationship with the concentration of H_2_O_2_, which provides a tool for inflammatory diseases imaging. Real-time imaging of healthy mice and acute lung injury/acute kidney injury with HP-H_2_O_2_ shows distinctly different fluorescence intensity (**Figure 4C-F**) 74, demonstrating the potential clinical utility. The work provides a convenient strategy to develop probes for various analytes and biomarkers via simple alkylation, acylation, or carbamylation of the N-methylamino attachment points. The balance between structure regulation and biocompatibility is a challenge for further application of these probes.

### RNS-activatable probes

RNS mainly include nitrogen dioxide (NO_2_), peroxynitrite (ONOO^-^), S-nitrosothiols (RSNO), nitric oxide (NO), and nitroxyl (HNO). RNS has a similar function to ROS in the organism 56, 75. The mechanisms related to the key RNS species have been employed to develop RNS-activatable probes.

ONOO^-^ is formed from nitric oxide and superoxide without enzymatic catalysis and can oxidize various biomolecules such as thiols, lipids, proteins, carbohydrates, DNA, and low-molecule weight anti-oxidants. Overproduction of ONOO^-^ would induce oxidative injury linked with a large number of diseases 75, 76. The sensing strategies of ONOO^-^-activatable probes include the production of amido from dearylation reaction (probe 14), deboronation (probe 15), oxidation of unsaturated bond (probe 16), and decomposition of α-ketoamide group (probe 17) and so on 77-80. NO can serve as an oxidative stress biomarker for diseases involving oxidative injury. The sensing strategies of NO-activatable probes are mostly based on the PET mechanism, including the generation of triazole the reaction from o-phenylenediamine (probe 18) and N-nitrosation reaction of aromatic secondary amine (probe 19) 81, 82.

In 2019, an ONOO^-^-activatable NIR-II molecular probe IRBTP-B was developed based on the incorporation of the phenyl borate group and a NIR-II fluorescence turn-on benzothiopyrylium cyanines skeleton (**Figure 5A**). Initially, the conjugated π-electron system is divided in half due to the masking of phenyl borate. In the presence of ONOO^-^, the phenyl borate group was removed to turn on the NIR-II fluorescence with a good linear response to ONOO^-^ (**Figure 5B-C**). IRBTP-B was used to monitor the upregulation of ONOO^-^ and the remediation with N-acetyl cysteine (NAC) with a preclinical drug-induced liver injury model *in vivo* (**Figure 5D-E**) 83. This work provides a strategy to design a variety of activatable NIR-II probes by masking the hydroxyl functionalized reactive site with an analyte-specific triggering group. Extending the emission to a longer wavelength (>1300 nm) may accelerate the clinical translation of the probe for drug-induced organism injury.

The same group reported an OONO^-^-activatable NIR-II probe PN1100 based on a panel of fluorescent dyes (CX) with chemo- and photo-stability (**Figure 5F-G**). PN1100 was designed by loading CX-1 and CX-3 into a micelle for OONO^-^ detection based on a FRET mechanism between CX-1 and CX-3 dyes (**Figure 5H**). Upon treatment with OONO^-^ (0-24 μM), the ratio of F_920_/F_1130_, attributed to the CX-1 and CX-3, increased exponentially due to the oxidation of CX-3 (**Figure 5I-J**). The *in vivo* detection of drug-induced hepatotoxicity with PN1100 was conducted by using APAP-treated mice as a model. The ratios of the APAP-treated group were higher than those of the PBS-treated and remediation (APAP+NAC) groups, which confirms the generation of OONO^-^ in the liver of APAP-treated mice (**Figure 5K**) 84. CX dyes show great application potential in bioimaging due to their promising spectral properties and ease of synthesis. However, the development of analogs with a larger Stokes shift and further FRET-based applications are the challenges for future work.

In 2019, a NO-activatable organic semiconducting nanoprobe was fabricated, which was designed by FTBD and a polymer (styrene-co-maleic anhydride) (PSMA) with good water solubility by amidation (**Figure 6A**). The AOSNP turns on its second near-infrared window fluorescence by using a NO-sensitive organic semiconducting group (FTBD) for its primary sensing component. When being exposed to NO, FTBD can transform its energy acceptor units (benzo[c][1,2,5]thiadiazole-5,6-diamine) into stronger acceptors (benzotriazole derivatives) to redshift the fluorescence (**Figure 6B**). Furthermore, the fluorescence intensity ratio F/F_0_ (where F_0_ indicates the fluorescence intensity of the AOSNP without NO and F is the fluorescence intensity in the presence of the indicated concentration of NO) showed a good linear relationship (R^2^ = 0.9961) with NO concentration within various NO concentrations (0-35 µM) (**Figure 6C**). Based on its excellent response, the AOSNP has been successfully used for *in vivo*, *in situ*, real-time, and non-invasive NIR-II fluorescence monitoring of drug dose-dependent NO activity associated with hepatotoxicity, specifically for the commonly used anti-pain/fever drug APAP (**Figure 6D**) 85. This work shows the potential of the NO-activatable probe in the detection of hepatotoxicity. However, designing probes with a longer emission wavelength and higher biocompatibility based on the strategy is a challenge for clinical application.

### RSS-activatable probes

RSS is active as antioxidants and signaling agents in a variety of tissue types, including the liver, gastrointestinal system pancreas, brain, and circulatory system, which contribute to many physiological responses to maintain cellular health 56, 86. RSS contain thiols (GSH, Cys, Hcy), disulfide (RSSR), persulfides (R-S-SH/H_2_S_2_), H_2_S, sulfenic acid (RSOH), thiyl radical (RS·), polysulfides (R_2_Sn/H_2_Sn, n > 2), and sulfur dioxide/sulfite/bisulfite (SO_2_/SO_3_^2-^/HSO^3-^) 56, 87. Abnormal levels of RSS may contribute to many diseases such as Down syndrome, Alzheimer's disease, cirrhosis, diabetes, and cancer-based on previous reports 62, 63. The mechanisms related to some key RSS have been explored for the development of RSS-activatable probes.

The production and metabolism of H_2_S in mammalian cells are regulated by enzymes that are distributed throughout virtually every tissue type. Monitoring the dynamics of H_2_S *in vivo* provides a better understanding of basic physiological and pathological mechanisms. Based on the nucleophilic and reducing capacity of H_2_S, the sensing strategies include nucleophilic addition reaction (probe 20), H_2_S-induced thiolysis (probe 21), the reduction of azide group (probe 22), etc. 88-90. As a vital biomarker, GSH plays a key role in maintaining physiological homeostasis. Based on the nucleophilic capacity of GSH, the sensing strategies of the GSH-activatable NIR-II probes are mainly based on nucleophilic substitution or Michael addition reaction (probe 23, 24) 91, 92. In general, the activatable probes are designed based on the nucleophilicity of the RSS.

The ratiometric NIR-II H_2_S-responsive nanoprobes NIR-II@Si were devised, which are comprised of an H_2_S-responsive BODIPY (ZX-NIR) dye and an H_2_S-inert aza-BODIPY (aza-BOD) dye for the internal reference (**Figure 7A**). Upon the H_2_S stimuli, the NIR-II fluorescence of ZX-NIR at 900-1300 nm was lighted up due to the transformation of ZX-NIR into NIR-II-HS through aromatic nucleophilic substitution. Meanwhile, aza-BOD has poor overlapping with ZX-NIR and NIRII‐HS and chemical inertness to H_2_S, providing an ideal inference (**Figure 7B**). This target-specific activatable probe was used for visualization of colorectal cancers (CRC) *in vivo* in HCT116 subcutaneous xenograft nude mice (**Figure 7C**) 86. The ratiometric probe provides a novel idea for the development of smart NIR-II activatable probes. Improving the loading percentage of responsive agents in the nanocomposites and improving biocompatibility are the challenges for further application. Subsequently, the same group developed an H_2_S-activatable nanostructured photothermal agent (Nano-PT) for site-specific NIR-II fluorescence-guided photothermal therapy of CRC (**Figure 7D**). The Nano-PT not only emits NIR-II fluorescence in the presence of H_2_S (**Figure 7E**) but also generates high NIR absorption, resulting in efficient photothermal conversion under NIR laser irradiation. The *in vivo* treatment outcome of the photothermal therapy with Nano-PT shows good tumor inhibition (**Figure 7F**) 93. This work shows the potential of NIR-II responsive probes for photothermal therapy. Improving the biocompatibility of self-assemble nanoparticles can accelerate further application.

The NIR-II H_2_S-responsive probes WH-X with the D-π-A structure were fabricated by introducing 4-Nitrothiophenol into the difluoro BODIPY fluorescent probe as the fluorescence quencher and specific recognition site. WH-3 was selected as the probe for H_2_S imaging due to the balance of long emission (1020-1400 nm, centered at 1140 nm) and acceptable QY (0.17% in PBS) (**Figure 7G**). NIR-II fluorescence intensity centered at 1140 nm enhanced when WH-3 was treated with an increasing concentration of NaHS (**Figure 7H**), which can be attributed to the enhanced intramolecular charge-transfer process. Imaging results of mice show the fluorescence intensity of tumor sites increased along with tumor size amplification associated with increased H_2_S levels (**Figure 7I**) 94. WH-3 was successfully applied to real-time monitoring of endogenous H_2_S generation and fluctuation in tumor-bearing mice. Improving the QY of activatable probes based on this flexible strategy is the key to the further application.

The NIR-II GSH-responsive probes LET-7 was composed of an anionic polymethylcyanide dye linked with a quenching group, 3,5-bis(trifluoromethyl)benzenethiol (BTBT). In the presence of GSH, LET-7 was transformed into turn-on signal reporter LET-G due to the replacement of the BTBT with GSH (**Figure 8A**). LET-7 showed the specific response to GSH and exhibited a perfect linear relationship with the concentration of GSH, which provides a tool for related diseases imaging (**Figure 8B-C**). A 4T1 tumor-bearing mouse model was established for the *in vivo* NIR-II FL monitoring of GSH. The result showed that the brightness and SBR of tumor regions are higher than those in normal tissue regions, which showed the application potential of LET-7 (**Figure 8D-E**) 95. This novel design strategy can provide ideas for the detection of other disease-related biomarkers. However, extending the NIR-II emission to a longer wavelength and developing more stable probes based on the polymethylcyanide structure are the challenges for further applications.

## Microenvironment

**Figure 9:**
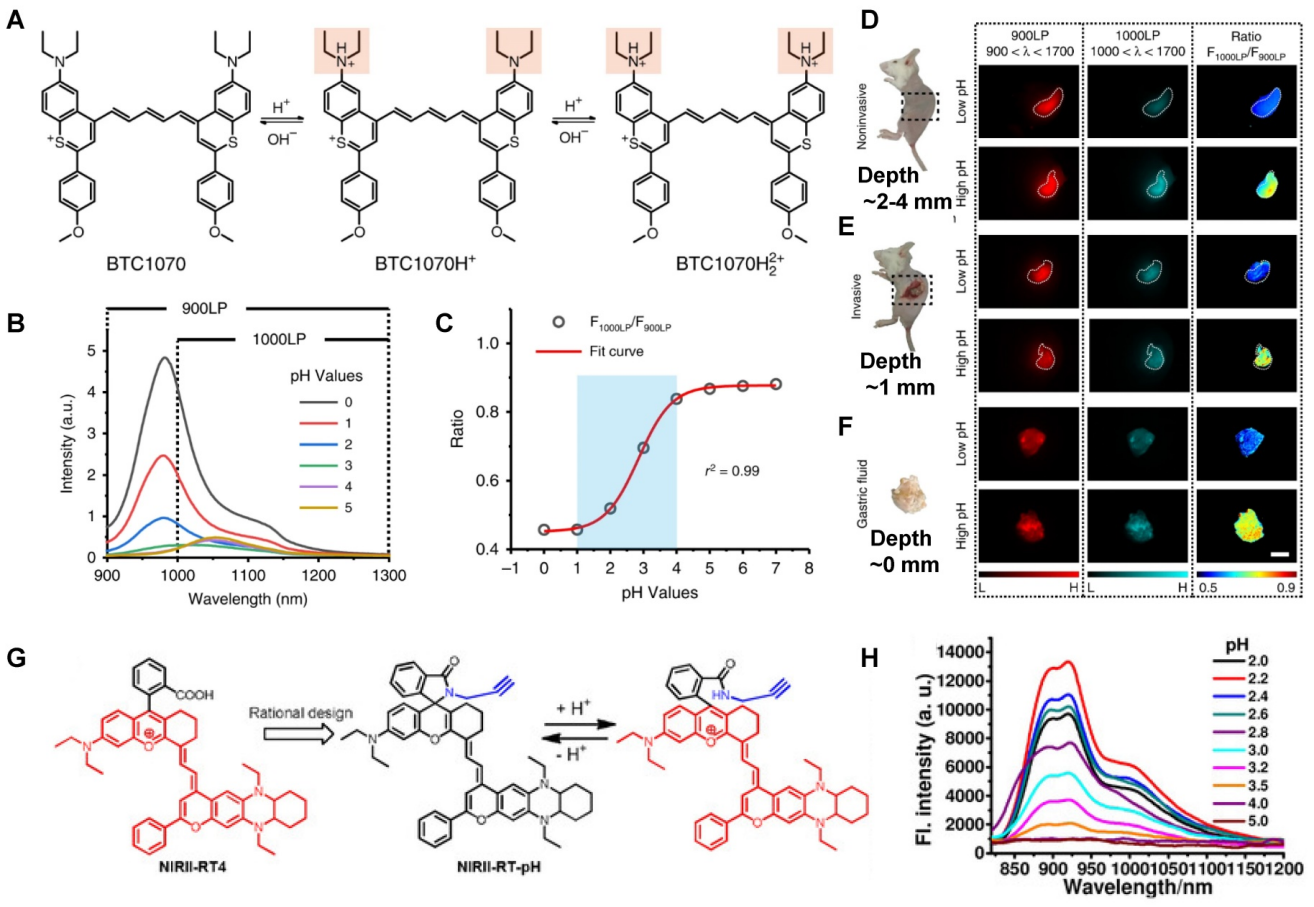
** Activatable NIR-II probes for pH. (A)** The structure and detection mechanism of BTC1070 for pH. **(B)** The fluorescence spectra at various pH values under the excitation of 808 nm. **(C)** Plot of fluorescence ratio changes as a function of pH values. Ratio = F_1000LP_/F_900LP_, F_1000LP_ and F_900LP_ denote the integrated intensity at 1000-1300 nm and 900-1300 nm, respectively. **(D-F)** Left: digital photographs of mice and dissected stomach. Right: fluorescence images and corresponding ratiometric fluorescence images of mice stomachs at two different pH environments. **(G)** The structure and detection mechenisem of NIRII-RT-pH. **(H)** The fluorescence spectra of NIRII-RT-pH at various pH values. Figures adapted with permission from 110, 111. Copyright © 2019, Springer Nature, and Copyright © 2020, John Wiley and Sons.

**Figure 10:**
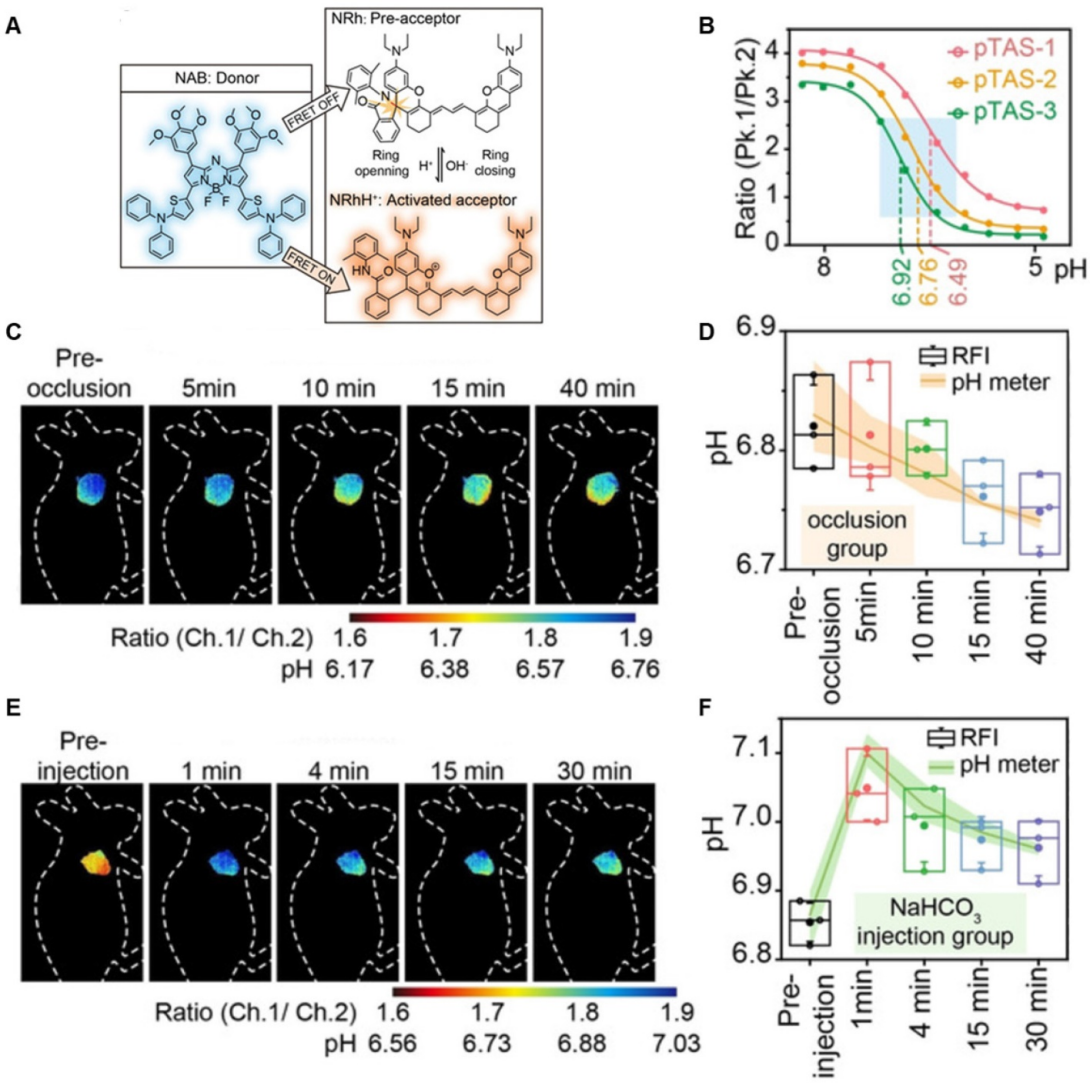
** Activatable NIR-II probes for pH. (A)** The structure and detection mechanism of pTAS. **(B)** Fluorescence intensity ratio (F_940 nm_/ F_1026 nm_) of four pTAS as a function of pH. The ratiometric fluorescence images of tumors during the two pH changing processes after administration of pTAS-2 **(C)** and pTAS-3 **(E)**, respectively. **(D)** and **(F):** Corresponding comparison of the pH changing in two processes by ratiometric fluorescence imaging and microelectrode pH meter. Figures adapted with permission from^100^. Copyright © 2021, John Wiley and Sons.

**Figure 11:**
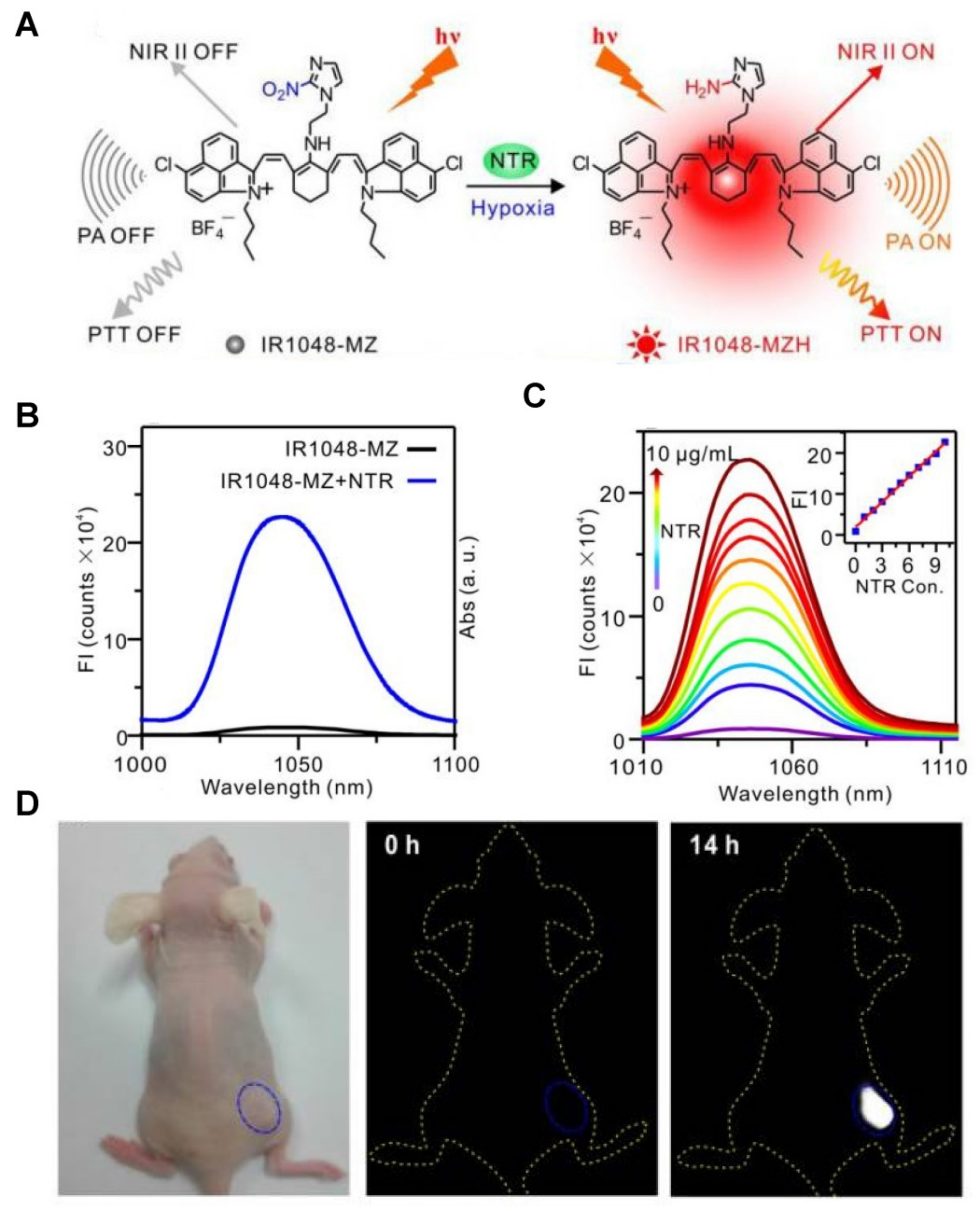
** Activatable NIR-II probes for various hypoxia. (A)** Probing hypoxia using IR1048-MZ conjugating a nitroimidazole group with an IR-1048 dye. **(B)** Fluorescence emission spectra (λex/λem = 980/1046 nm) in the absence and presence of NTR. **(C)** NIR-II fluorescence responses of IR1048-MZ (5 µg/mL) to different concentrations of NTR. Inset shows a linear correlation between emission intensity and concentration of NTR. **(D)** NIR-II fluorescence imaging of tumors in living mice at the highest-signal time. Figures adapted with permission from 115. Copyright © 2018, Ivyspring International.

**Figure 12:**
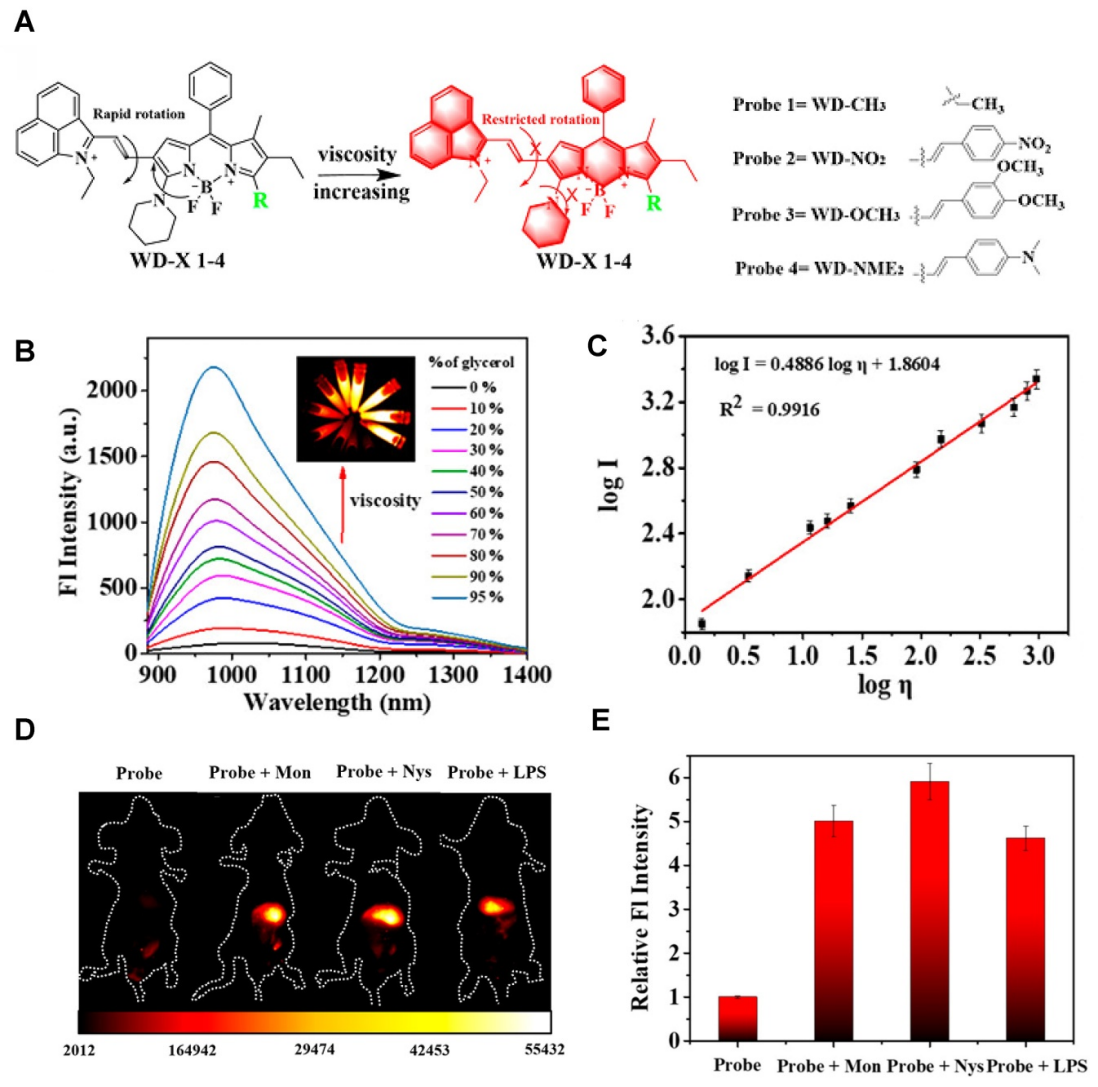
** Activatable NIR-II probes for viscosity. (A)** The structure of WD-X and its mechanism for detecting viscosity, which is based on BODIPY skeleton binding with electron-drawing (nitro) or electron-donating groups. **(B)** Fluorescence spectra of WD-NO_2_ (20 µM) in various ethanol-glycerol mixture. Inset shows corresponding fluorescence images of WD-NO_2_. **(C)** The linear relationship between the logarithmic fluorescence intensity of WD-NO_2_ at 982 nm and log η. **(D)** Fluorescence imaging of viscosity changes by exogenous drug stimulation. Mice were only intraperitoneally injected with WD-NO_2_. Mice were intraperitoneally injected with Mon, Nys, and LPS accompanied with intraperitoneal injection of WD-NO_2_, respectively. **(E)** Corresponding fluorescence intensity of mice (Excitation: 808 nm laser, 50 mW cm^-2^). Figures adapted with permission from 121. Copyright © 2020, American Chemical Society.

Tissue microenvironment factors such as pH, viscosity and oxygen pressure play a key role in maintaining the metabolism and normal physiological function. On the condition that the microenvironment varies, it can alter the metabolism and even remodel the tissue leading to pathological changes and proceeding to disease. For another, pathological changes also can result in microenvironment factors alteration. Therefore, the inspection of microenvironment factors can not only be a method for early diagnosis but also provide clues for pathogenesis 96-98, and a variety of responsive organic NIR-II fluorescence probes were developed recently (**Table 1 and Figure 9-12**).

### pH-activatable probes

The normal intracellular pH (pHi) level of adult cells is ~7.2 and extracellular pH (pHe) is ~7.4. pHi plays a critical role in normal physical activity such as ion transport, multidrug resistance (MDR) and proliferation, apoptosis. Abnormal pH values are usually associated with cellar dysfunction, leading to cancers or neurodegenerative disorders. For example, cancer cells have reverse pH levels of pHi > 7.4 and pHe ~6.7-7.1. Lowered extracellular pH is an important signature strongly associated with cancer invasion, progression, and metastasis. However, its malignant effects were mainly identified using cell and tissue studies 99, 100. Thus, monitoring pH levels in living organs is critical for providing information on the physiological and pathological processes 101-104.

pH-activatable probes are mostly designed based on the cyanine (probe 25), BODIPY (probe 26), coumarin (probe 27), naphthalimide (probe 28), and rhodamine skeletons (probe 29) 105-109. BODIPY derivatives can be used as common pH-activatable probes due to their excellent emission and photostability. By introducing various groups at corresponding sites, the fluorescent dyes can gain new emissions and realize pH sensing. Cyanine- and hemicyanine-based probes can realize pH sensing by the protonation and deprotonation of the pH sensing site. Rhodamine serves as a natural pH sensor for developing a variety of probes due to the acid-controlled spiro structure. Generally, other fluorescent groups like cyanine, BODIPY and coumarin participate in the energy transfer process of rhodamine probes, thus modulating the emission to realize the pH sensing.

Gastric acid is a key factor in maintaining normal upper gastrointestinal function and can be used as a valuable biomarker. In 2019, a series of benzothiopyrylium pentamethine cyanines BTCs were reported by introducing electron-donating diethylamino moieties to overcome the solvatochromism-caused quenching and achieve stable NIR-II emission. The selected BTC1070 with the longest wavelength shows pH-responsive properties in an aqueous solution. When the pH values changed from 5 to 0 (**Figure 9B**), the blue-shifted fluorescence and enhanced intensity were observed under 808 nm excitation. The excellent responsive pH range is optimized to 1-4 from the sigmoidal equation plot of fluorescence ratio at the wavelength of 1000-1300 nm and 900-1300 nm (**Figure 9C**). The protonation mechanism is proved to be protonation on nitrogen atoms, resulting in ratiometric fluorescence response to various pH through the inhibition of ICT effect (**Figure 9A**). Based on the above property, ratiometric imaging *in vivo* was conducted for quantification of gastric pH, showing good agreement with measurement by standard pH meter. Noninvasive NIR-II imaging at ~ 2-4 mm tissue depth in the BTC1070-injected mice shows a clear stomach profile and significant difference in pseudo-color at two pH environments (**Figure 9D-F**) 110. This work provides a strategy for pH-activatable probes based on the modulation of the π-electron system of benzothiopyrylium or other heterocycles. Developing brighter probes with longer emission wavelength is the challenge for further application.

In 2020, a new class of polymethine dyes NIRII-RTs were designed to serve as effective NIR-II platforms for the development of activatable bioimaging probes by introducing the carboxylic acid functional group. NIRII-RT-pH shows increasing fluorescence intensity at 925/1000 nm with the pH regulated from 4.0 to 2.2 (**Figure 9G-H**). A series of target-activatable NIRII-RT probes are successfully applied to the NIR-II imaging, confirming the feasibility of this novel design stratagem 111. This work unlocks the potential of cyanine fluorophores and provides bright design ideas for target-activatable probes.

A series of pH transition-point adjustable sensors (pTAS) are developed by regulating the component ratio of NIR-II emission aza-BODIPY donor (NAB) and pH activatable rhodamine-based acceptor (NRh) in FRET system (**Figure 10A**). With pH changing from 8.30 to 4.88, the fluorescence intensity of pTAS at 940 nm declined obviously, while the fluorescence emission at 1026 nm was observed (**Figure 10B**). Thereinto, pTAS-1~3, the fluorescence intensity ratio of Peak 1 (940 nm) and Peak 2 (1026 nm) at pH 8.30-4.88 show satisfied pH response ability with well-fitted sigmoidal Boltzmann functions. pTAS-1~3 have the corresponding pH response regions 6.11-6.88 (ΔpH=0.77), 6.43-7.09 (ΔpH=0.66), and 6.63-7.22 (ΔpH=0.59), respectively. More importantly, the combination of three pH response regions provides a twofold widened pH detection range of 6.11-7.22, which is regulated by the NAB-NRh molar ratio. pTAS-2 and pTAS-3 are successfully applied to tumor pH monitoring, illustrating invasive vascular occlusion-induced lactic acid accumulation at the tumor site and the buffering ability of fluid in the tumor (**Figure 10C-F**) 100. This work provided a novel FRET design strategy for pH-activatable probes with a widened pH detection region of 6.11-7.22 and may accelerate its clinical application.

### Hypoxia-activatable probes

Hypoxia is a feature of most tumors and exhibits chemo- or radiation resistance unfavorable for cancer therapies 112-114. Hypoxia causes the overexpression of nitroreductase (NTR), thus the quantitative detection of NTR can be used to evaluate the tumor hypoxic degree. The hypoxia-activatable single-molecule probe IR1048-MZ was designed by conjugating an electron-with drawing group (2-(2-nitroimidazolyl) ethylamine, MZ) with a NIR-II dye IR-1048 (**Figure 11A**) 115. The MZ group of IR1048-MZ was reduced to the corresponding amine group in the presence of NTR, leading to the inhibition of electron transfer and the emerging NIR-II fluorescence and PA signal of IR1048-MZ (**Figure 11B**). The fluorescence intensity at the peak wavelength of 1046 nm excited by 980 nm increased linearly with the increasing NTR concentration (0-10 μg/mL) (**Figure 11C**). Furthermore, the detection limit value of NTR was found to be 43 ng/mL. The NIR-II fluorescence imaging *in vivo* was conducted in bearing subcutaneous A549 tumor mice to observe the response to tumor hypoxia. IR1048-MZ showed the highest NIR-II fluorescence signal at 14 h, meanwhile, no obvious background signal was observed in the process (**Figure 11D**). Moreover, the hypoxia-activated photothermal therapy of IR1048-MZ showed outstanding phototherapy efficacy with rapid temperature increases. Improving the biocompatibility of IR1048-MZ can facilitate the further clinical application.

### Viscosity-activatable probes

Viscosity is a key indicator of biological microenvironments related to various physiological and pathological processes. Monitoring the levels of viscosity can provide critical information for related diseases 116, 117. The developed viscosity-activatable probes show various strategies, including interactions between the targeting group and the skeleton (probe 30), conjugation effect of D-π-A structure (probe 31), fluorescent molecular rotors based on fluorescence lifetime imaging (probe 32) etc. 118-120. The targeting groups are usually the lysosomal and mitochondria localization group like morpholine, pyridinium cations, and the skeletons are usually the Cy, BODIPY, coumarin, and rhodamine. The ICT, FRET and PET effects of these probes are controlled by viscosity, thus regulating fluorescence intensity for sensing.

A series of viscosity-activatable NIR-II emissive probes WD-X were designed and synthesized for tracing the variation of viscosity in diabetes-induced liver injury *in vivo*, screening the most optimized WD-NO_2_. The probes are designed by connecting the BODIPY dye modified with different groups and 1-ethyl-2-methyl-benz[c,d] iodolium salt with vinyl bond. The vinyl bond, as well as the C-N single bond of the BODIPY moiety of WD-X, can't rotate freely at high viscosities, resulting in strong fluorescence intensity due to the favorable conjugate structure (**Figure 12A**). With the increase of viscosity (the glycerol content increasing from 0 to 95%), WD-NO_2_ showed a 31-fold fluorescence intensity enhancement at 982 nm. Meanwhile, the relationship between the fluorescence intensity of WD-NO_2_ and viscosity (1.52-925 cP) is consistent with the Forster-Hoffmann linear equation: log I = 0.4886 log η + 1.8604 (R^2^ = 0.9916) (**Figure 12B-C**). This quantitative relationship endows visualization of the variation of viscosity in diabetes-induced liver injury *in vivo*. The fluorescence intensity was enhanced in mice treated with monensin (Mon), nystatin (Nys), and lipopolysaccharide (LPS), respectively, compared with the control group injected with probe only (**Figure 12D-E**) 121. This work firstly reported a NIR-II emissive viscosity-activatable fluorescent probe through a novel structure design, which gives more ideas for the development of viscosity-activatable molecular probes in the NIR-II region. Improving the biocompatibility and extending the NIR-II emission to a longer wavelength are the challenges for further application.

## Enzyme-activatable probes

**Figure 13:**
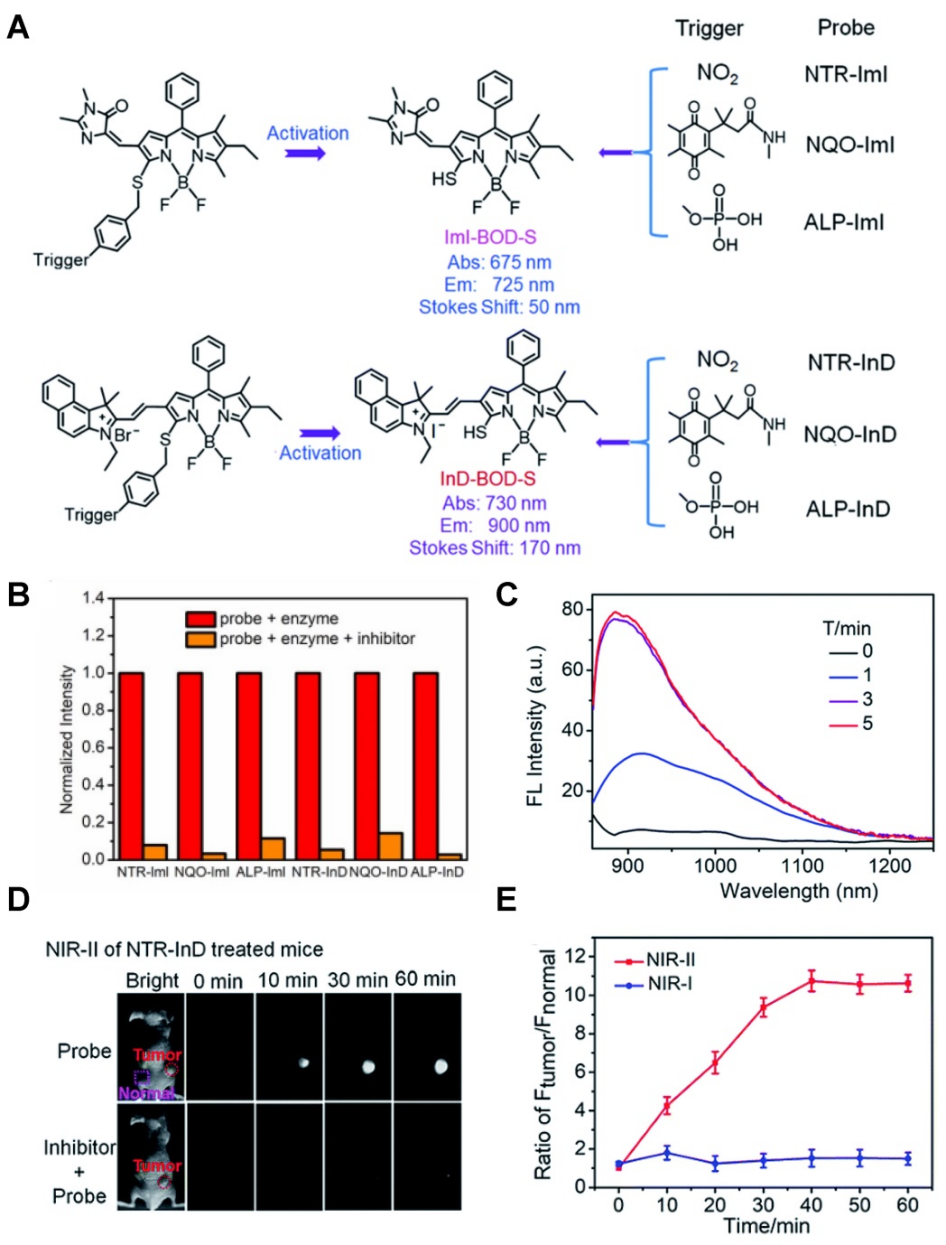
** Activatable NIR-II probes for NTR. (A)** Detection mechanism of the probe for NTR, NQO1, or ALP by conjugating BODIPY with enzymic substrates via a self-immolative benzyl thioether linker. **(B)** Enzymes-induced the fluorescence intensity changes of probes in the absence or presence of inhibitors. **(C)** NIR-II fluorescence spectra of NTR-InD upon addition of NTR (20 µg mL^-1^) in buffer. **(D)** Time-dependent NIR-II imaging of mice injected with NTR-InD (30 nmol) or NTR-InD + dicoumarol (0.3 mmol). **(E)** The fluorescence intensity of the tumor in NIR-I imaging and NIR-II imaging via the region of interest analysis. Figures adapted with permission from 129. Copyright © 2010, Royal Society of Chemistry.

**Figure 14:**
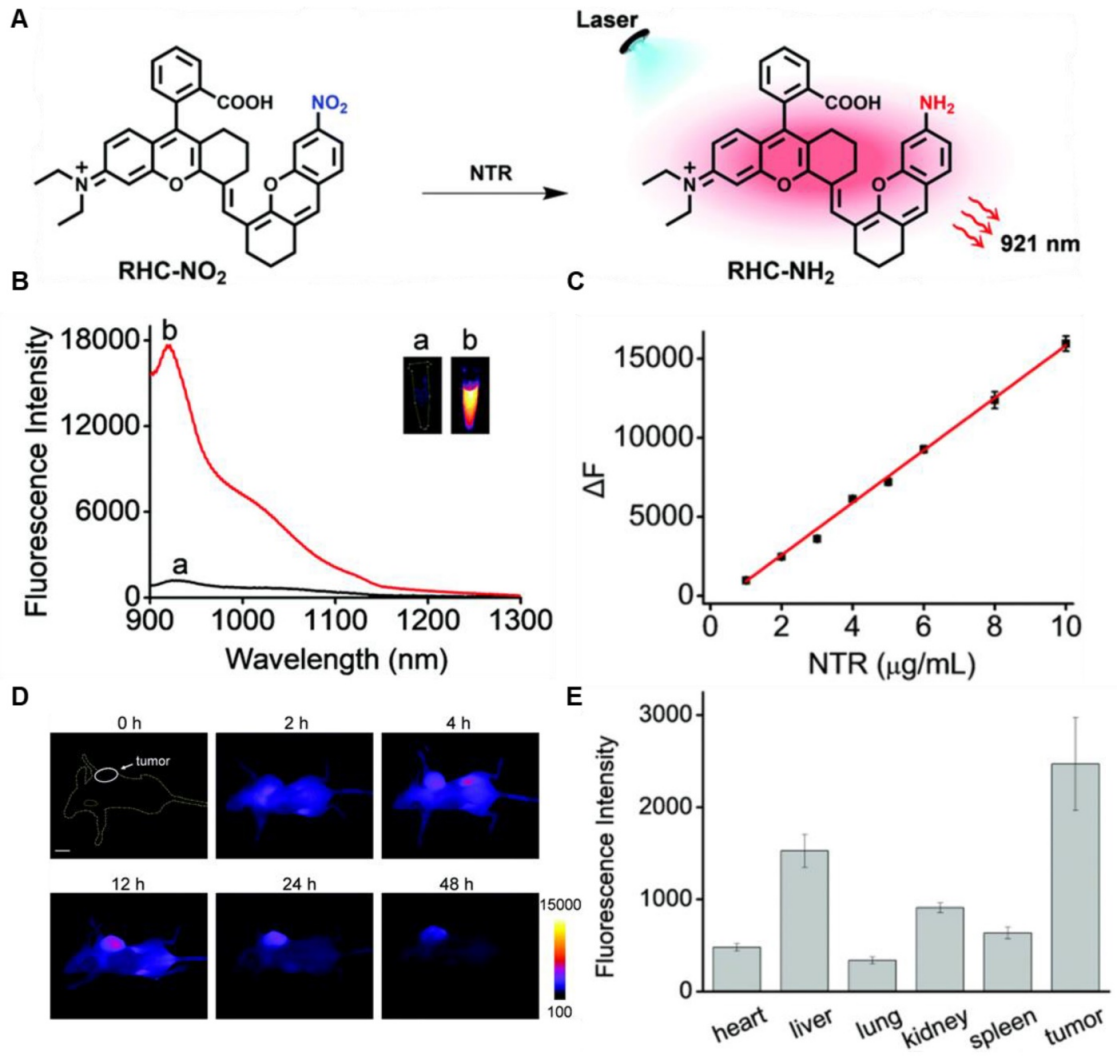
** Activatable NIR-II probes for NTR. (A)** The structure and mechanism of RHC-NO_2_ for NTR. **(B)** The fluorescence spectra of RHC-NO_2_ with and without NTR. **(C)** The linear fitting plot of ΔF against the concentration of NTR.** (D)** NIR-II images of A549 tumor-bearing mice after injection of RHC-NO_2_. **(E)** Quantified fluorescence intensity of dissected organs and the tumor. Figures adapted with permission from 130. Copyright © 2021, Royal Society of Chemistry.

**Figure 15:**
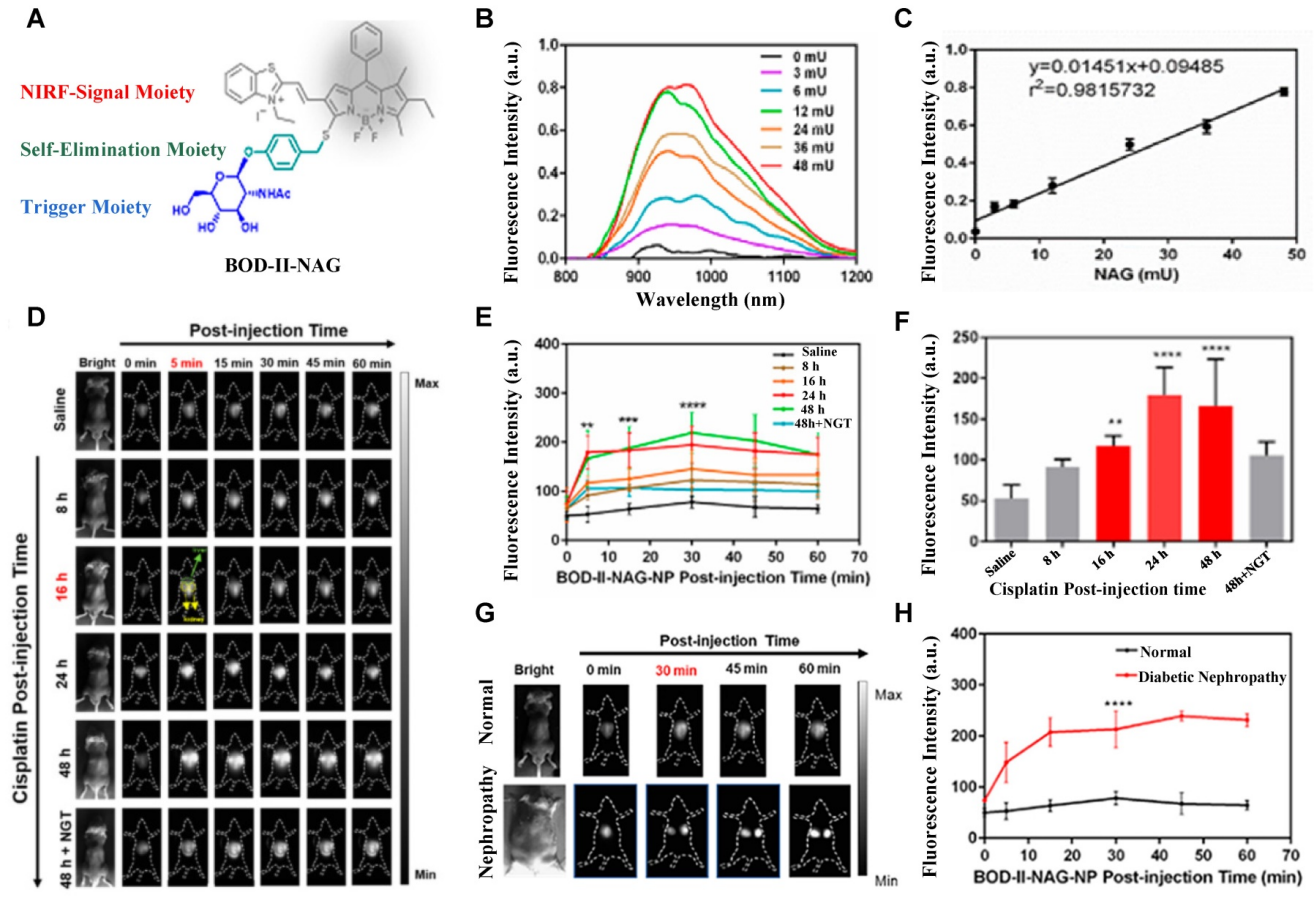
** Activatable NIR-II probes for NAG. (A)** Illustration of structure and inspection mechanism of BOD-II-NAG for NAG, based on BODIPY skeleton conjugated with N-acetyl-β-d-glucosamine residues. **(B)** Fluorescence spectral changes of and BOD-II-NAG (10 µM) with increasing concentration of NAG with 30 min incubation at 37 °C. **(C)** Plots of the linear relationship between fluorescence intensity at 1000 nm and concentrations of NAG. **(D)** Real-time NIR-II fluorescence imaging of the control group, the AKI group, and the inhibitor group mice after intravenous injection of BOD-II-NAG-NP (16 µM/kg body weight). **(E)** Corresponding real-time NIR-II fluorescence intensities of kidneys and **(F)** quantitative results. **(G)** Real-time NIR-II fluorescence imaging of with or without diabetic nephropathy mice after intravenous injection of BOD-II-NAG-NP. **(H)** Corresponding real-time NIR-II fluorescence intensities of kidneys. Figures adapted with permission from 133. Copyright © 2021, American Chemical Society.

The abnormal expression of enzymes is a promising biomarker of tumors, and enzyme-activatable probes were developed harnessing the merits of enzymes such as high catalytic efficiency and specific substrate selectivity 122-124 (**Table 1 and Figure 13-15**). Based on the high activity and specificity of the enzyme, the strategy of enzyme-activatable probes is based on the reaction of the substrates and the enzyme. The activatable probes for nitroreductase (NTR), quinone oxidoreductase isozyme 1 (NQO1), alkaline phosphatase (ALP), γ-Glutamyltransferase (l-GGT), etc. have been developed, and each type of probe has its corresponding recognition site. The structures of NTR, ALP, NQO1, γ-GGT-activatable probes are designed upon corresponding substrates of p-nitrobenzyl moiety (probe 33), phosphate group (probe 34), quinone moiety (probe 35), γ-glutamyl group, conjugated (probe 36) with fluorophores, respectively 125-128.

The diverse enzyme-activated fluorescent probes were reported, which are designed by combining BODIPY platforms with enzymic substrates using a self-immolative benzyl thioether linker. These NIR probes are nitroreductase (NTR), quinone oxidoreductase isozyme 1 (NQO1), or alkaline phosphatase (ALP) probes by different modifications (**Figure 13A**), exhibiting specific enzyme detection (**Figure 13B**). The fluorescence intensity of NTR-InD is 12-fold increased under the treatment of NTR (**Figure 13C**). The NIR-II imaging of A549 subcutaneous xenograft nude mice injected with NTR-InD shows a high tumor-to-normal tissue ratio from the tumor-derived enzyme (**Figure 13D-E**) 129. Furthermore, the NIR-II signal detected by NTR-InD has deeper detection and higher T/N than the NIR-I signal by NTR-ImI, showing the potential *in vivo* tumor imaging of NIR-II activatable probes. The coupling of BODIPY platforms with different enzymic substrates in this work provides a novel design strategy for enzyme-activatable fluorescence probes. However, optimization of the water solubility by using the supramolecular assembly or hydrophilic groups is the challenge for further work.

In 2021, an NTR-activatable NIR-II fluorescence probe RHC-NO_2_ was developed based on the rhodamine hybrid polymethine framework and a nitro group as recognition moiety and was successfully applied in fluorescence imaging of tumors (**Figure 14A**). After the reaction of RHC-NO_2_ with NTR, 14-fold enhanced fluorescence was produced at 921 nm. RHC-NO_2_ showed a good linear relationship with the concentration of NTR, which can be applied to detect the concentration of NTR in the NIR-II window (**Figure 14B-C**). RHC-NO_2_ was successfully applied in the fluorescence imaging of two kinds of murine tumor models (A549 and HeLa tumors), and tumor margins were clearly observed (**Figure 14D-E**) 130. This work provides a convenient way to delineate the tumor margins for related diagnosis and treatment research. However, the low QY limits further clinical application, and further research may focus on the long emission wavelength and high QY.

N-acetyl-β-d-glucosaminidase (NAG) received considerable attention due to its clinical implications as a sensitive and specific biomarker for renal diseases 131, 132. In 2021, A NAG-activatable probe BOD-II-NAG is developed by incorporating N-acetyl-β-d-glucosamine residues into BODIPY-based NIR fluorescent probes via a self-elimination linker (**Figure 15A**). In the presence of NAG, BOD-II-NAG turns on NIR-II fluorescence response at 1000 nm due to the hydrolyzation of N-acetyl-β-d-glucosamine residues from probes catalyzed by NAG. BOD-II-NAG displayed increasing fluorescence intensity treated with increasing NAG concentrations, which shows a perfect linear relationship (**Figure 15B-C**). *In vivo* NIR-II imaging was conducted on acute kidney injury (AKI) and chronic kidney mice (CKD) for tracking NAG activity. In the cisplatin-induced AKI imaging, BOD-II-NAG showed obvious NIR-II signals in the early stage of the AKI due to the reduced autofluorescence, high sensitivity, and rapid response (**Figure 15D-F**). In the diabetic nephropathy CKD imaging, BOD-II-NAG showed more obvious and clearer NIR-II signals in the CKD group than the control group (**Figure 15G-H**) 133. The imaging results indicated that BOD-II-NAG has the potential to diagnose AKI or CKD. This work shows the huge advantage of activatable NIR-II probe BOD-II-NAG compared with the NIR-I probe BOD-I-NAG. Improving the biocompatibility of the probe may be helpful for the earlier diagnosis of related diseases *in vivo*.

## Dually-activatable probes

**Figure 16:**
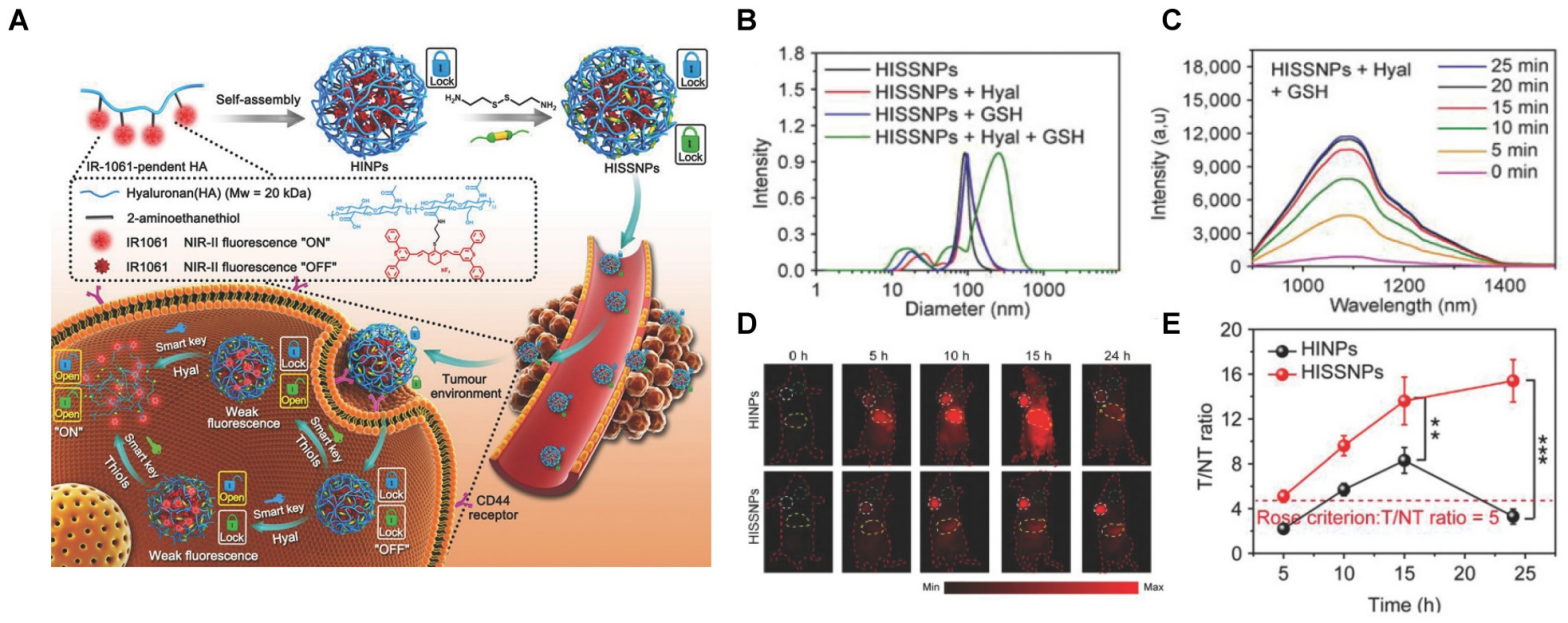
** Activatable NIR-II probes for hyaluronidase and thiols. (A)** Fabrication of HISSNPs and the response process for Hyaluronidase and Thiols, which is based on the crosslink of IR-1061-pendent HA polymers (HINPs) and disulfide. **(B)** DLS changes of and HISNPs in the absence or presence of Hyal and/or GSH. **(C)** Time-dependent fluorescence spectra of Hyal (0.1 mg mL^-1^) and GSH (1 × 10^-3^ M) in PBS (pH = 7.4) at 37 °C excited at 808 nm. **(D)** Real-time NIR-II fluorescence imaging of cancer mice injected with HISSNPs and HINPs. The white circle indicates the tumor site. The yellow circle indicates the abdominal liver site. The muscle in the green circle corresponds to the normal tissue region used to calculate the T/NT ratio. **(E)** Corresponding quantitative T/NT ratio in tumor mice at 24 h post-injection of HISSNPs and HINPs. Figures adapted with permission from 142. Copyright © 2018, John Wiley and Sons.

**Figure 17:**
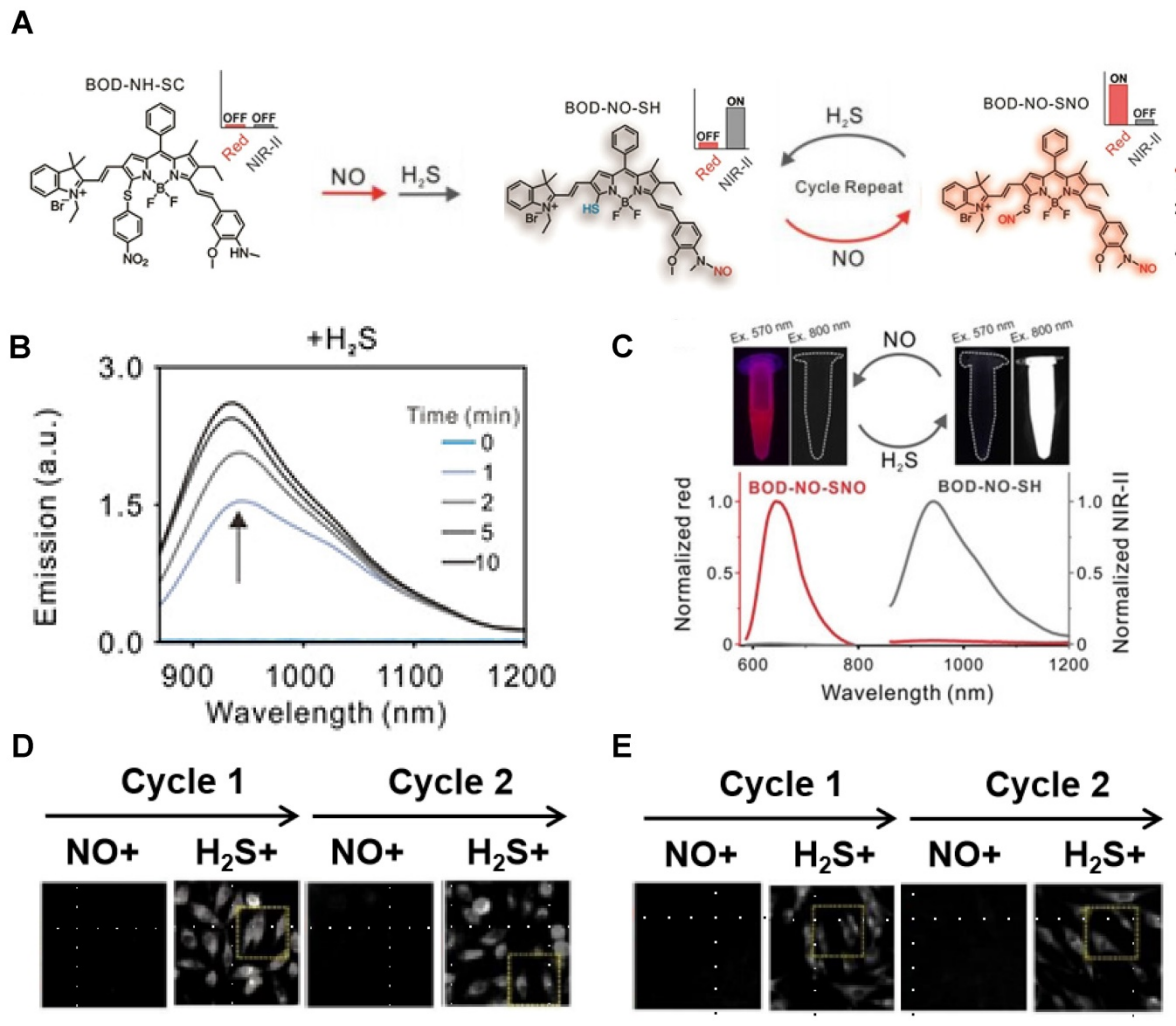
** Activatable NIR-II probes for NO and H_2_S. (A)** The structure of BOD-NH-SC and the response process on NO and H_2_S. **(B)** Time-dependent fluorescence spectra of BOD-NH-SC (5 µM) in the presence of H_2_S (100 µM). **(C)** Fluorescence spectra varying with cycled S-nitrosation and transnitrosation processes. Imaging in NIR-II fluorescence channel of **(D)** HepG2 and **(E)** colonic smooth muscle cells in the alternating presence of NO and H_2_S. Figures adapted with permission from 143. Copyright © 2021. John Wiley and Sons.

**Figure 18:**
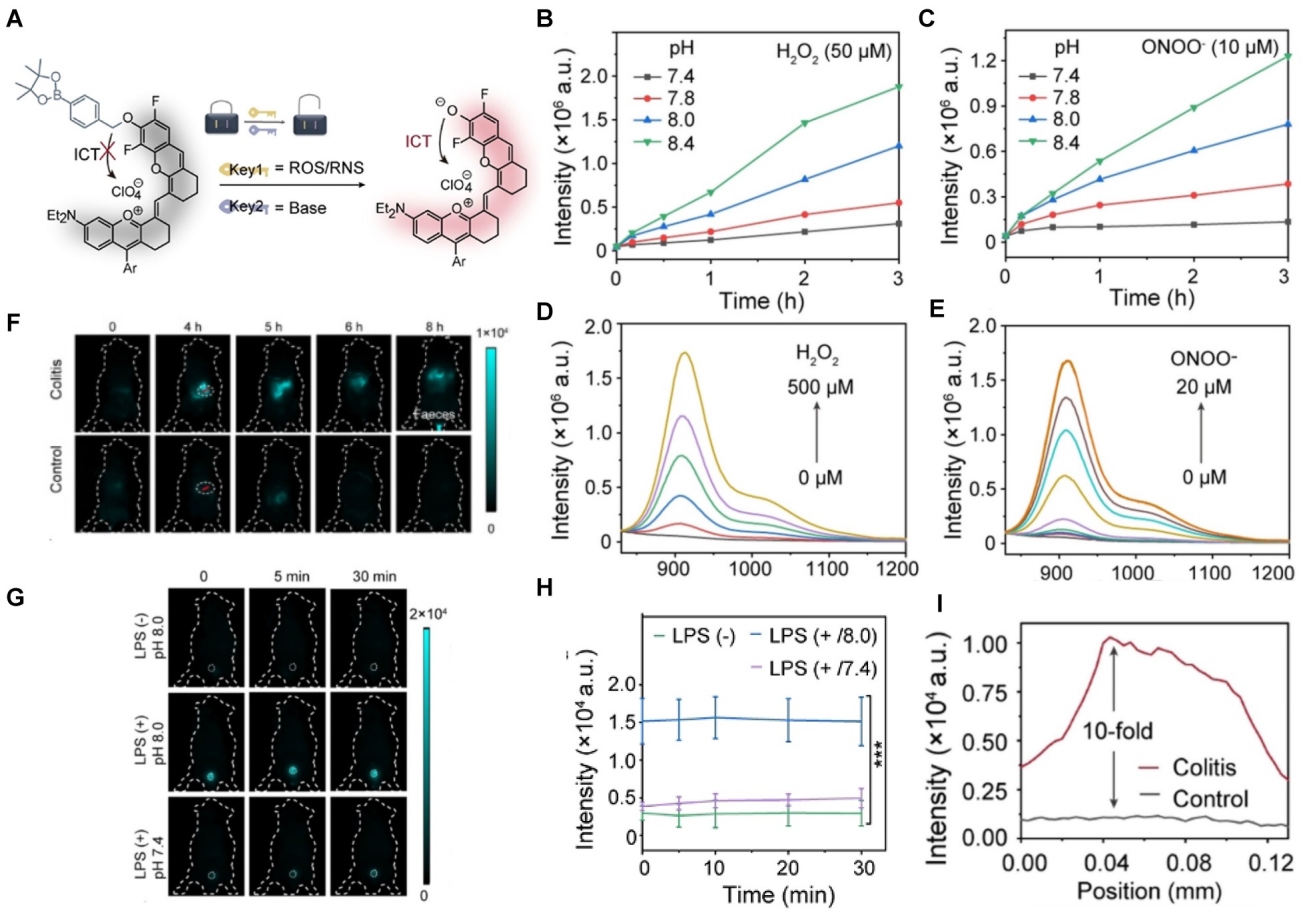
** Activatable NIR-II probes for ROS/RNS and base. (A)** The structure of PN910 and its detection mechanism for ROS/RNS and base. Time-dependent fluorescence intensity at 910 nm of PN910 (10 µM) with different pH values in the presence of **(B)** H_2_O_2_ and **(C)** ONOO^-^. Emission changes of PN910 in the presence of different concentrations of **(D)** H_2_O_2_ and **(E)** ONOO^-^. **(F)** Real-time imaging of colitis mice and healthy mice (Excitation: 808 nm laser, 30 mW cm^-2^) and **(I)** corresponding fluorescence intensity in the interest region. **(G)** Real-time imaging of normal mice and cystitis mice (Excitation: 808 nm laser, 30 mW cm^-2^) and **(H)** corresponding time-dependent fluorescence intensity of bladder. Figures adapted with permission from 146. Copyright © 2018, John Wiley and Sons.

Responsive probes associated with a single molecule are easy to emit “false positive” signals, while probes activated by two or more pathological changes make the imaging precise and become more attractive (**Table 1 and Figure 16-18**) 102, 134-141. An activatable NIR-II nanoprobe HISSNPs was developed, which responds to dual-pathological-parameter to control the fluorescence condition for tumor imaging. Meanwhile, the “dual-lock-and-key” probe with more accurate results is designed for the first time. The HISSNPs was constructed by following steps. Firstly IR-1061, hydrophobic cyanine dyes, and HA-hydrophilic hyaluronic acid were used to form the amphipathic HA polymers, further assembled into “single lock-and-key” nanoparticles HINPs. Furthermore, the “dual-lock-and-key” activatable HISSNPs was designed by crosslink of added disulfide with the HA of HINPs. The HISSNPs turn on its NIR-II fluorescence in the presence of hyaluronidase and thiols (**Figure 16A**). The DLS shows particle size of HISSNPs changed from monodispersity to polydisperse in the presence of Hyal and GSH, while no obvious changes under the single stimulus (**Figure 16B**). The HISSNPs shows the extremely enhanced signal in the presence of Hyal and GSH (**Figure 16C**), combing with the DLS results to confirm the correlation between the fluorescence intensity and the aggregation degree of IR-1061. The “dual lock-and-key” probe HISSNPs exhibits about fivefold higher greater tumor-to-normal tissue ratio than “single-lock-and-key” nanoparticles HINPs, achieving a better resolution between tumor region and normal tissues (**Figure 16D-E**) 142. However, improving the water solubility and biocompatibility of HISSNPs is the challenge for further work.

A dually responsive probe BOD-NH-SC was reported, which applies an N-Methyl-2-methoxyaniline moiety for NO-responsive site and 4-nitrobenzenethiol-substituted BODIPY for H_2_S-responsive site (**Figure 17A**). BOD-NH-SC emits bright NIR‐II fluorescence after being treated with successive NO and H_2_S, resulting in the formation of BOD-NO-SH. BOD-NO-SH shows cycled S‐nitrosation in the presence of NO and subsequent transnitrosation processes initiated by H_2_S, which is in the form of repeated colors changing from NIR-II fluorescence at 936 nm to NIR fluorescence at 645 nm (**Figure 17B-C**). Both colonic smooth muscle and HepG2 cells treated with BOD-NH-SC show bright fluorescence signals in the red channel (650-660 nm) with minimal fluorescence in the NIR-II channel (900-1000 nm) in the NO-pretreated process, while increasing fluorescence signals in the NIR-II channel with diminishing signal in the red channel in the presence of NaHS for H_2_S-initiated reaction (**Figure 17D-E**) 143. The results confirm the visualization of the dynamic condition of NO and H_2_S in living cells with a single fluorescent probe for the first time. Meanwhile, extending the emission to a longer wavelength can boost the clinical translation.

Acid-base balance maintains basic life activities, especially for normal urology and digestion 101, 144, 145. In 2021, a ROS/RNS and base dual activatable NIR-II molecular probe PN910 was developed based on modulating the ICT effect of a merocyanine fluorescent probe (**Figure 18A**). PN910 shows an increasing fluorescence intensity with the increase of H_2_O_2_ and ONOO^-^ at pH 8.0, while weak fluorescence in a neutral environment (**Figure 18B-E**). This is favorable for the imaging of alkaline microenvironments, such as the urine and the colon. Thus, LPS-induced cystitis and DSS-induced colitis were studied by *in vivo* imaging of PN910. In colitis imaging, the fluorescence signal of acute colitis mice was 10-fold higher than the control group mice (**Figure 18F, I**). In cystitis imaging, a brilliant fluorescence signal was observed in the pH 8.0 environment compared with neglected fluorescence the control groups (pH 7.4 with LPS injection, pH 8.0 without LPS injection) were nearly non-fluorescent (**Figure 18G-H**) 146. Imaging results confirmed that PN910 was a reliable probe for the monitoring of colitis and cystitis through* in vivo* imaging.

## Conclusions and perspective

Activatable NIR‐II fluorescence probes hold great promise in detecting disease-related biomarkers with high resolution and fast response 32, 147-149, and show potential in clinical translation. Besides, several aspects ought to be taken into account when design the responsive NIR-II fluorescence probes to boost the development and clinical translation.

First, biocompatibility is critical for clinical approval, thus the stability and metabolic property should be considered when designing the probes 150. Especially, for the nanostructures, controlling the hydrodynamic sizes below 5.5 nm can drive them excreted via the urinary system to decrease cumulative toxicity and benefit their potential clinical applications 151-153. In addition, the water solubility of organic fluorescent probes is important for their metabolism, thus hydrotropic groups can be introduced to modify the fluorescent probe skeleton.

Second, the photoluminescence properties, especially high QY, should be reckoned 154-163. Based on the photochemical mechanisms such as PET 164-167, FRET 168-170, ICT 171-173, fluorescent probes with long emission wavelengths will be developed continuously. Additionally, the QY and photochemical stability are critical for the usefulness of the fluorescent probes. To date, a structure-function relationship that can guide the design to improve the QY has not been established, while structural modification of aromatic systems that possess high QY tends to be a useful strategy to design activatable organic fluorescent probes. The photodegradation of organic fluorescence probes can follow different oxygen-dependent or oxygen-independent mechanisms, and involve such parameters as fluorophore structure, medium conditions, irradiation power. The main strategy for enhancing the photostability is to improve the design of fluorophores, particularly to attach protective and anti-fading groups to the molecular nuclear. For instance, shielding units were designed to protect the D-A-D structure and can improve the photochemical stability of the organic probes 174, 175. With high QY and photochemical stability, the activatable fluorescence probes are promising for bioimaging.

Third, improving targeting is another aspect to be considered for developing activatable NIR-II fluorescence probes, and several strategies such as binding with targeting molecules, introducing extracorporeal stimulus, and designing dual or multiple responsive reactivities may motivate the development 21, 176-180. In addition, probes based on fluorescence lifetime imaging provide novel strategies for the development of activatable probes 181.

Fourth, the theranostic design of activatable NIR-II fluorescent probes is an important approach for diseases treatment. For instance, besides the photon down conversion and photothermal conversion, exploring novel energy conversion modes could provide new ideas for tumor theranostics in the NIR-II region. Converting the energy into oxidation stress through the low-energy NIR-II photon excitation is a strategy for therapeutic purposes 150.

In conclusion, a long-term effort is still needed to address the challenges and the NIR-II fluorescence probes exhibit promising prospects for rapid development and clinical translation.

## Figures and Tables

**Table 1:** Representative organic responsive NIR-II fluorescence probes

Probes	Structure	Analyte	Photochemical designed Mechanism	Absorbance [nm]	ex [nm]	em [nm]	Imaging applications	Detection limit	Biocompatibility	t_1/2_	SNR	QY	Ref
Hydro-1080	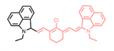	•OH	Target-induced change in the chemical structures of fluorescent probes	1021	1064	1044	Monitor •OH induced by LPS and APAP overdose	0.5 nM	The results of the MTT assay toward HeLa cells show low cytotoxicity of Hydro-1080 (0-20 μM)		6.0	0.45%	73
SPNP25	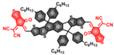	ClO^-^	Bio-erasable intermolecular donor-acceptor interaction	700	808	1000-1700	*In vivo* dynamic inflammation sensing	0.68 μM	The results of *in vivo* toxicology show no obvious long toxicity in the mice treated with SPNP25 (50 µg)	~2.5 h	17.5	0.21%	72
HP- H_2_O_2_	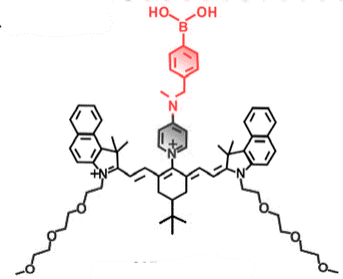	H_2_O_2_	Photoinduced electron-transfer	826	808	937	ALI and AKI imaging		The results of histology show no liver-toxicity in the mice treated with HP- H_2_O_2_ (50 µM)	~1 h		0.30%	74
IRBTP-B	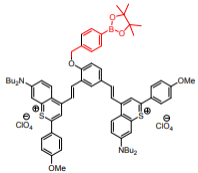	ONOO^-^	Target-induced change in the chemical structures of fluorescent probes	600	808	1100	APAP-induced hepatotoxicity monitoring	55.9 nM	The results of histology show no liver toxicity in the mice treated with IRBTP-B (1mM)		5	0.10%	83
PN1100	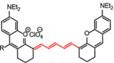	ONOO^-^	FRET	1089	808	F_920_/ F_1130_	APAP-induced hepatotoxicity monitoring		The results of histology show no liver toxicity in the mice treated with PN1100		17.4	0.091±0.014	84
AOSNP	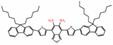	NO	Transforming weak acceptors of fluorescent probes into strong acceptors to shift the fluorescence	680	808	1000-1700	APAP-induced hepatotoxicity monitoring	0.35 μM	The results of MTT assay show low cytotoxicity of AOSNP (200 μg/mL)	~1.5 h		0.35%	85
NIR-II@Si	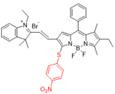	H_2_S	Target-induced change in the chemical structures of fluorescent probes	520	780	900	The identification of colon cancer cells and differentiation between types of living cells	37 nM	The results of *in vivo* biodistribution and pharmacokinetic studies show low toxicity in the mice treated with NIR-II@Si (20 nmol ZX-NIR)		5.7	0.37 %	86
Nano-PT	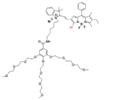	H_2_S	Target-induced change in the chemical structures of chromophores	540, 790	785		Photothermal therapy of CRC	106 nM	The results of histopathology and blood biochemistry show low toxicity in the mice treated with Nano-PT (100 nmol SSS)	~6.5 h	8.3±0.5	0.0034%	93
WH-3	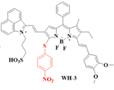	H_2_S	ICT	575, 925	925	1140	Real-time Monitoring of endogenous H_2_S generation and fluctuation in tumor-bearing mice	51 nM	The results of *in vivo* toxicology showed that WH-3 has superior biocompatibility and biosafety (100 μL, 1.0 mM)		10.64	0.17%	94
LET-7	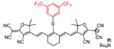	GSH	Target-induced change in the chemical structures of fluorescent probes	900	808	928	Real-time visualization of GSH in tumors	85 nM	The results of *in vivo* toxicology showed that LET-7 has excellent biocompatibility (50 μL, 10 μM)		7.5		95
BTC1070	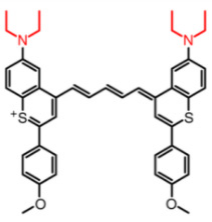	pH	ICT	1015, 950	808	F_1000_/F_900_	Noninvasive ratiometric quantification of gastric pH	1-4	The results of the cytotoxicity assay show high cell viability of BTCs (40 μM)		9.42	0.016%	110
NIRII-RT-pH	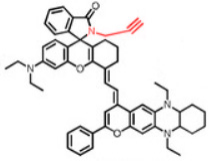	pH	Target-induced change in the chemical structures of fluorescent probes	856		925	Real-time monitoring of drug-induced hepatotoxicity		The results of the MTT assay toward HeLa cells show low cytotoxicity of NIRII-RTs (0-10 μM)			1.42%	111
pTAS	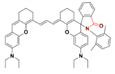	pH	FRET	895	808	940, 1026	*In vivo* dynamic tumor pH monitoring	6.11-7.22				2.08%	100
IR1048-MZ	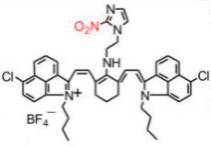	Hypoxia	Target-induced change in the chemical structures of fluorescent probes	980	980	1046	Hypoxia-activated photothermal cancer therapy	43 ng/mL	The results of *in vivo* toxicology show high biocompatibility of IR1048-MZ (40 μg/mL)		30	0.006%	115
NTR-InD	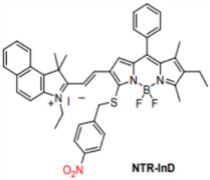	NTR	Target-induced change in the chemical structures of chromophores	553	730	1000-1300	Monitoring of enzyme activities for Targeted cancer cell imaging and differentiation		The probes show low cytotoxicity toward living cells		10.6	3.9%	129
RHC-NO_2_	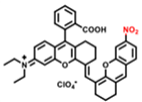	NTR	Target-induced change in the chemical structures of fluorescent probes	677	808	921	NIR-II tumor imaging	5.9 ng/mL	The results of the MTT assay toward A549 and HeLa cells show low cytotoxicity of RHC-NO_2_ (0-100 μM)			<0.01	130
BOD-II-NAG-NP	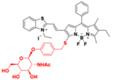	NAG	The hydrolyzation of N-acetyl-β-d-glucosamine residues	490	710	1000	Tracking the activity of NAG *in vivo* and exploring its potential role in AKI or CKD diagnosis.	0.72 mU / mL	The results of the cytotoxicity assay show low cytotoxicity of BOD-II-NAG-NP (0-40 μM)		15	0.002%	133
WD-NO_2_	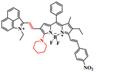	Viscosity	Target-induced change in dihedral angle and conjugated region	768	818	982	Track the variation of viscosity in diabetes-induced liver injury		The results of the MTT assay toward HeLa cells show very low cytotoxicity of WD-NO_2_ (0-30 μM)		4.4	0.230%	121
HISSNPs	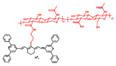	Hyaluronidase and thiols	Target-induced change in aggregation state	810, 1050	808	1000-1700	Ultrahigh specific imaging of tumor *in vivo*		The results of *in vivo* toxicology show low toxicity in the mice treated with HISSNPs (35 mg/kg)		15.4		142
BOD-NH-SC	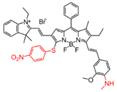	NO and H_2_S	Aromatic nucleophilic substitution	664	808	936	Monitoring the alternating existence of NO and H_2_S in living cells	20 nM	The results of the MTT assay show low cytotoxicity of BOD-NH-SC (0-1 μM)			0.06%	143
PN910	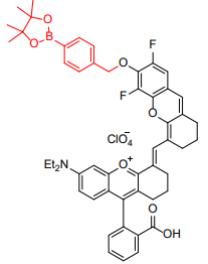	ROS/RNS and base	ICT	675	808	910	Monitoring cystitis and colitis	1 μM (ONOO^-^)	The results of *in vivo* toxicology show little toxicity in the mice treated with Chrodol-3 (10 mg/kg)		10		146

The red part on each fluorophore represents the reaction moiety that can be activated by the analytes and enable the molecular luminescence.
